# K-OPLS package: Kernel-based orthogonal projections to latent structures for prediction and interpretation in feature space

**DOI:** 10.1186/1471-2105-9-106

**Published:** 2008-02-19

**Authors:** Max Bylesjö, Mattias Rantalainen, Jeremy K Nicholson, Elaine Holmes, Johan Trygg

**Affiliations:** 1 Research Group for Chemometrics, Department of Chemistry, Umeå University, Umeå, SE-901 87, Sweden; 2 Department of Biomolecular Medicine, Division of Surgery, Oncology, Reproductive Biology and Anaesthetics (SORA), Faculty of Medicine, Imperial College, London, SW7 2AZ, UK

## Abstract

**Background:**

Kernel-based classification and regression methods have been successfully applied to modelling a wide variety of biological data. The Kernel-based Orthogonal Projections to Latent Structures (K-OPLS) method offers unique properties facilitating separate modelling of predictive variation and structured noise in the feature space. While providing prediction results similar to other kernel-based methods, K-OPLS features enhanced interpretational capabilities; allowing detection of unanticipated systematic variation in the data such as instrumental drift, batch variability or unexpected biological variation.

**Results:**

We demonstrate an implementation of the K-OPLS algorithm for MATLAB and R, licensed under the GNU GPL and available at . The package includes essential functionality and documentation for model evaluation (using cross-validation), training and prediction of future samples. Incorporated is also a set of diagnostic tools and plot functions to simplify the visualisation of data, e.g. for detecting trends or for identification of outlying samples. The utility of the software package is demonstrated by means of a metabolic profiling data set from a biological study of hybrid aspen.

**Conclusion:**

The properties of the K-OPLS method are well suited for analysis of biological data, which in conjunction with the availability of the outlined open-source package provides a comprehensive solution for kernel-based analysis in bioinformatics applications.

## Background

Orthogonal Projections to Latent Structures (OPLS) [1,2] is a linear regression method that has been employed successfully for prediction modelling in various biological and biochemical applications [3-5]. Among the benefits provided by the OPLS method is its innate ability to model data with both noisy as well as multi-collinear variables, such as spectral data from metabolic profiling and other omics platforms [6]. The OPLS method employs the descriptor matrix **X **(*N *× *K*), where *N *denotes the number of samples and *K *the number of variables in **X**, to predict the response matrix **Y **(*N *× *M*), where *M *denotes the number of variables in **Y**. The unique property of the OPLS method compared to other linear regression methods is its ability to separate the modelling of co-varying variation from structured noise, defined as systematic **Y**-orthogonal variation, while simultaneously maximising the covariance between **X **and **Y**.

The OPLS algorithm models the variation in the data matrix **X **by means of two sets of latent variables [7] (score matrices) **T**_p _and **T**_o_; see Equation 1. Here, **T**_p _(*N *× *A*) denotes the **Y**-predictive score matrix for **X**, **P**_p_^T ^(*A *× *K*) denotes the **Y**-predictive loading matrix for **X**, **T**_o _(*N *× *A*_o_) denotes the corresponding **Y**-orthogonal score matrix, **P**_o_^T ^(*A*_o _× *K*) denotes the loading matrix of **Y**-orthogonal components and **E **denotes the residual matrix of **X**. Both the **Y**-predictive and **Y**-orthogonal score matrices describe properties of the modelled observations that are useful for identifying expected and unexpected trends, clusters or outlying samples in data. The relationship between OPLS and other linear regression methods is discussed explicitly elsewhere [1,3].

(1) **X **= **T**_p_**P**_p_^T ^+ **T**_o_**P**_o_^T ^+ **E **

Kernel-based pattern recognition methods [8] such as Support Vector Machines (SVMs) [9], Kernel-PCA (KPCA) [10,11] and Kernel-PLS (KPLS) [12,13] have previously been applied in a multitude of contexts for exploratory analysis and classification, including biological applications [14-17]. Common among these kernel-based methods is their application of the 'kernel trick' [18]; allowing the kernel matrix to be treated as dot products in a high-dimensional feature space. Specifically, this is achieved by adopting a linear method to so-called dual form, so that all instances of the descriptor matrix **X **are expressed in terms of dot products, e.g. **XX**^T^. Subsequently, **XX**^T ^is substituted for the kernel Gram matrix **K **with entries **K**_*i*,*j *_= k(**x**_*i*_, **x**_*j*_), where **x**_*i *_and **x**_*j *_corresponds to the *i*th and *j*th row-vector in the descriptor matrix **X**, respectively, and k(·,·) represents the kernel function. Hence, one can avoid explicitly mapping **X **to higher-dimensional spaces as well as computing dot products in the feature space, which is computationally beneficial. The transformation to higher-dimensional spaces is performed implicitly by the kernel function k(·,·); where common kernel functions include polynomial or Gaussian functions (see Equations 2 and 3).

(2) k(**x**,**y**) = (**x**^T^**y **+ 1)^*p *^

(3) k(**x**,**y**) = exp(-||**x**-**y**||
^2^/2*σ*^2^)

The kernel functions in Equations 2–3 depend on the choice of the parameters *p *and *σ*, respectively, which typically influences the predictive ability of the kernel-based method. The traditional approach to kernel parameter selection is to pre-define parameter limits and subsequently perform an exhaustive grid search over the entire parameter space. At each setting, the generalisation properties of the model are evaluated using e.g. cross-validation [19] to identify the parameter setting yielding the lowest possible generalisation error. Unfortunately, even moderately short step sizes can result in a large number of evaluations and unacceptable run times. The alternative in such cases is to utilise stochastic methods, such as simulated annealing [20], which may identify reasonable approximations of the global generalisation error minimum using less evaluations.

The Kernel-OPLS method [21] is a recent reformulation of the original OPLS method to its kernel equivalent. K-OPLS has been developed with the aim of combining the strengths of kernel-based methods to model non-linear structures in the data while maintaining the ability of the OPLS method to model structured noise. The K-OPLS algorithm allows estimation of an OPLS model in the feature space, thus combining these features. In analogy with the conventional OPLS model, the K-OPLS model contains a set of predictive components **T**_p _and a set of **Y**-orthogonal components **T**_o_. This separate modelling of **Y**-predictive and **Y**-orthogonal components does not affect the predictive power of the method, which is comparable to KPLS and least-squares SVMs [22]. However, the explicit modelling of structured noise in the feature space can be a valuable tool to detect unexpected anomalies in the data, such as instrumental drift, batch differences or unanticipated biological variation and is not performed by any other kernel-based method to the knowledge of the authors. Pseudo-code for the K-OPLS method is available in Table 1. For further details regarding the K-OPLS method, see Rantalainen *et al*. [21].

**Table 1:** Pseudo-code for the K-OPLS model training algorithm. K denotes the original kernel matrix, K_i _the kernel matrix deflated by *i *Y-orthogonal components and Q_i _the K_i _matrix deflated by *A *predictive components.

**Step**	**Description**	
1.	Estimate the predictive **Y**-weights (**C**_p_) by eigen-vector decomposition of **Y**^T^**KY**	
2.	Project **Y **onto **C**_p _to achieve the predictive score matrix of **Y**: **U**_p _← **YC**_p_	
3.	Calculate the predictive score matrix of **X**: **T**_p _← **KU**_p_	
4.	Repeat for *i *: 1 to *A*_o_	
	4.1	Estimate the **Y**-orthogonal loadings **c**_o _by eigen-vector decomposition of **T**_p_^T^**Q**_i_**T**_p_.
	4.2.	Calculate the **Y**-orthogonal score vector: **t**_o,i _← **Q**_i_**T**_p_**c**_o_
	4.3.	Deflate **K**_i _by **t**_o,i_, yielding **K**_i+1_
	4.4.	Update the predictive score matrix: **T**_p _← **K**_i+1_**U**_p_
5.	Predictions of **Y**: **Y**_hat _← T* _p _(**T**_p_^T^**T**_p_)^-1^**T**_p_^T^**U**_p_**C**_p_^T^. For predictions of future samples, T* _p _originates from the prediction set.	

Implementations of various kernel-based methods are available in the literature for the R and MATLAB environments. Among the R packages available on CRAN [23], a few relevant examples include kernlab (kernel-based regression and classification), e1071 (including SVMs) and PLS (implementing a linear kernel-based implementation of the PLS algorithm). kernlab provides a number of kernel-based methods for regression and classification, including SVMs and least-squares SVMs, with functionality for *n*-fold cross-validation. The e1071 package contains functions for training and prediction using SVMs, including (randomised) *n*-fold cross-validation. The PLS package includes an implementation of both linear PLS as well as a linear kernel-based PLS version. This enables more efficient computations in situations where the number of observations is very large in relation to the number of features. The PLS package also provides a flexible cross-validation functionality.

MATLAB toolboxes implementing kernel-based methods include e.g. the SVM and Kernel Methods MATLAB Toolbox [24], Least Squares – Support Vector Machines MATLAB/C toolbox [25] and libsvm [26]. The latter contains a general collection of SVM related algorithms implemented in C++ and Java, including interfaces for MATLAB, Python and a number of other environments. All of these packages provide implementations of various kernel-based methods as well as cross-validation functionality and basic plot functions. Additional kernel-based software packages can be found at kernel-machines.org [27].

An implementation of the original linear OPLS method [1] is available in the Windows-based software SIMCA-P+ 11.0 (Umetrics AB, Umeå, Sweden). SIMCA-P includes a vast number of visualisation features as well as *n*-fold cross-validation functionality to estimate the number of **Y**-predictive and **Y**-orthogonal components.

Here, we describe an implementation of the K-OPLS algorithm for R [28] and MATLAB (The Mathworks, Natick, MA, USA) licensed under the GNU GPL. To the best knowledge of the authors, there are no other software packages currently available that implement the K-OPLS method. The package includes fundamental functionality for model training, prediction of unknown samples and evaluation by means of cross-validation. Included is also a set of diagnostic tools and plot functions to simplify the visualisation of data, e.g. for detecting trends or for identification of outlying samples.

The K-OPLS method can be used for both regression as well as classification tasks and has optimal performance in cases where the number of variables is much higher than the number of observations. Typical application areas are non-linear regression and classification problems using omics data sets. Properties of the K-OPLS method make it particularly helpful in cases where detecting and interpreting patterns in the data is of interest. This may e.g. involve instrumental drift over time in metabolic profiling applications using e.g. LC-MS or when there is a risk of dissimilarities between different experimental batches collected at different days. In addition, structured noise (**Y**-orthogonal variation) may also be present as a result of the biological system itself and can therefore be applied for the explicit detection and modelling of such variation. This is accomplished by interpretation of the **Y**-predictive and the **Y**-orthogonal score components in the K-OPLS model. The separation of **Y**-predictive and **Y**-orthogonal variation in the feature space is unique to the K-OPLS method and is not present in any other kernel-based method.

The utility of the K-OPLS software package is demonstrated by means of a metabolic profiling data set from a biological study of hybrid aspen, where the K-OPLS method is compared in parallel to the similar KPLS method.

## Implementation

The K-OPLS algorithm has been implemented as an open-source and platform-independent software package for MATLAB and R, in accordance with [21]. The K-OPLS package provides functionality for model training, prediction and evaluation using cross-validation. Additionally, model diagnostics and plot functions have been implemented to facilitate and further emphasise the interpretational strengths of the K-OPLS method compared to other related methods.

The following features are available for both MATLAB and R:

### (1) Estimation (training) of K-OPLS models

An implementation of the pseudo-code in Table 1 for modelling the relation between a kernel matrix **K **(*N *× *N*) and a response matrix **Y **using *A *predictive and *A*_o _**Y**-orthogonal score vectors.

### (2) Prediction of new data using the estimated K-OPLS model in step (1)

An implementation of the prediction of **Y**_hat _(*N*_*test *_× *M*) given a test kernel **K**_test _(*N*_*test *_× *N*_*test*_).

### (3) Cross-validation functionality to estimate the generalisation error of a K-OPLS model

This is intended to guide the selection of the number of **Y**-predictive components *A *and the number of **Y**-orthogonal components *A*_o_. The supported implementations are:

• *n*-fold cross-validation. Data is split into *n *separate groups and models are sequentially built from *n*-1 groups while the *n*th group is predicted and used to measure the generalisation error.

• Monte Carlo Cross-Validation (MCCV) [29]. Data is randomly split into cross-validation training and test sets. A model is built from the cross-validation training set while the test set is predicted and used to measure the generalisation error. The procedure is repeated *n *times to achieve a distribution of prediction errors.

• Monte Carlo Class-balanced Cross-Validation (for discriminant analysis cases). Same as regular MCCV except that the split into cross-validation training and test sets is balanced with respect to the existing class labels.

### (4) Kernel functions

including the polynomial (Equation 2) and Gaussian (Equation 3) kernel functions.

### (5) Model statistics

• The explained variation of **X **(R^2^_X_).

• The explained variation of **Y **(R^2^_Y_).

• Prediction statistics over cross-validation for regression tasks (Q^2^_Y_, which is inversely proportional to the generalisation error).

• Prediction statistics over cross-validation for classification tasks (sensitivity and specificity measures).

### (6) Plot functions for visualisation

• Scatter plot matrices for model score components.

• Model statistics and diagnostics plots.

Code examples for the functionality described above is available in Additional File 1 for both MATLAB and R. The K-OPLS package, including source code and documentation, is available for different operating systems in Additional Files 2, 3, 4 or for download on the project home page (see Availability and requirements).

## Results and Discussion

The utility of the method has previously been demonstrated using simulated data and for applications in analytical chemistry [21]. Here, we describe a biological data set originating from a study measuring differences in biochemical composition across two genotypes of hybrid aspen. The genotypes will be denoted *mutant *and *wild-type *(WT) throughout. Samples have been taken from three biological replicates of each genotype at eight different positions of the tree (internodes 1–8, starting from the top), constituting 48 different observations, of which 46 are included in the analysis (data collection failed for two samples). The internode gradient denotes an approximate growth gradient of the tree. Metabolic profiling data has been collected by means of high-resolution magic angle spinning proton nuclear magnetic resonance (^1^H HR/MAS NMR) spectroscopy. Data pre-treatment, including bucketing and removal of residual water, is described in the original study [30].

The modelled descriptor matrix **X **(46 × 655) contains the NMR data and the response matrix **Y **(46 × 1) contains the genotypes labelled as -1 and +1. The aim in this study is to predict an unknown sample into the correct category (mutant or WT) based on the metabolic profile. An additional 10 samples were used as an independent test set to further estimate the generalisation error. Both data sets were column-wise mean-centred prior to modelling.

**Figure 1:**
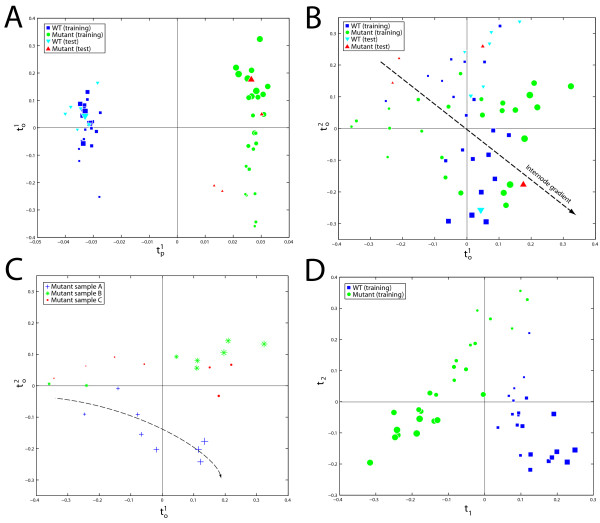
K-OPLS model properties of the NMR-based metabolic profiling data set. Each point represents a measured observation (biological sample).**The size of each glyph in the figure is proportional to the internode number 1–8, denoting a growth gradient**. In (**A**), the K-OPLS predictive score vector **t**^1^_p _is plotted against the first **Y**-orthogonal score vector **t**^1^_o_. In (**B**), the first K-OPLS **Y**-orthogonal score vector **t**^1^_o _is plotted against the second **Y**-orthogonal score vector **t**^2^_o_. An approximate joint internode gradient, formed by a linear combination of both vectors, is shown using the dashed arrow. In (**C**), the first K-OPLS **Y**-orthogonal score vector **t**^1^_o _is plotted against the second **Y**-orthogonal score vector **t**^2^_o _only for the mutant samples, colour-coded by biological replicate. Biological replicate A displays a deviating behaviour compared to biological replicates B and C; trajectory shown by the dashed line. In (**D**), the first KPLS latent variable **t**_1 _is plotted against the second latent variable **t**_2_. The discriminatory direction is now a linear combination of both of the latent variables.

A K-OPLS model was fitted using the Gaussian kernel function with σ = 0.5, one predictive component (**t**^1^_p_) and nine **Y**-orthogonal components (**t**^1–9^_o_) as recommended by seven-fold cross-validation. The model statistics R^2^_X _= 96.3%, R^2^_Y _= 100% and Q^2^_Y _= 93.6% (corresponding to 100% correct classifications during cross-validation) suggests a highly predictive and general model. The predictive score vector **t**^1^_p _is plotted against the first **Y**-orthogonal score vector **t**^1^_o _in Figure 1A. The discriminatory direction is described by **t**^1^_p_, showing that the classes are evidently well separated. From the external test set, which has been predicted into the model as shown in Figure 1A, all class labels of the test samples are correctly estimated. The **Y**-orthogonal components characterise variation that is systematic but linearly independent of the class labels. The variation in the first **Y**-orthogonal score vector **t**^1^_o _describes an internode (growth) gradient for the mutant samples but not for the WT samples, which is captured in **t**^2^_o _(Figure 1B). This implies that i) the internode gradients are systematic and independent of the direction separating the different genotypes; and ii) that the internode gradients are independent across the different genotypes. From a biological perspective, this is obviously an interesting effect induced in the mutant.

From Figure 1B one can also note that there is a joint internode gradient, formed by a linear combination of **t**^1^_o _and **t**^2^_o_. Furthermore, Figure 1B reveals a somewhat bimodal behaviour of the mutant internode gradient. In Figure 1C the joint internode gradient is shown only for the mutant samples, colour-coded by biological replicate. Biological replicate A displays a deviant behaviour, which is an intermediate between the profiles of biological replicates B and C and the WT samples (Figure 1B) and explains the bimodal behaviour. Also from the original study one can superficially see (Figure 4A on page 356 in [30]) that biological replicate A is an approximate intermediate of the stronger mutants B and C and the WT samples. A plausible explanation for this behaviour is that the anti-sense construct used to create the modified samples is not as strongly active in biological replicate A; either due to the process involved in generating the mutant or slight differences in growth conditions.

For comparison, a KPLS model was fitted in parallel using the Gaussian kernel function with σ = 0.5 and 10 **Y**-orthogonal components as recommended by seven-fold cross-validation. The first latent variable **t**_1 _is plotted against the second **t**_2 _in Figure 1D. One can note that the discriminatory direction is now a linear combination of both of the latent variables (and possible also subsequent components). The different internode gradients are distinctly seen also in the KPLS model, although the internode gradient of the WT samples is correlating perfectly with the discriminatory direction, implying that this direction is related to the class separation. In relation to the K-OPLS model, one can clearly see that this is not the case from Figure 1A–B and previous discussions, which highlights the advantages of the K-OPLS method. Furthermore, it is not possible in the KPLS model to quantify the amount of variance related to class discrimination (34.3% from the K-OPLS model) in relation to the variance related to the internode gradient (47.3% based on the variance in **t**^1^_o _and **t**^2^_o _in the K-OPLS model).

Practical code examples of the functionality of the package are available in Additional File 1, describing both MATLAB and R code including illustrations from an additional demonstration data set. This demonstration data set also is available with the supplied package (Additional Files 2, 3, 4).

## Conclusion

Kernel methods have previously been applied successfully in many different pattern recognition applications due to the strong predictive abilities and availability of the methods. The K-OPLS method is well suited for analysis of biological data, foremost through its innate capability to separately model predictive variation and structured noise. This property of the K-OPLS method has the potential to improve the interpretation of biological data, as was demonstrated by a plant NMR data set where interpretation is enhanced compared to the related method KPLS. In conjunction with the availability of the outlined open-source package, K-OPLS provides a comprehensive solution for kernel-based analysis in bioinformatics applications.

## Availability and requirements

• **Project name**: kopls

• **Project home page**: 

• **Operating systems: **OS Portable (Source code to work with many OS platforms).

• **Programming languages**: MATLAB and R

• **Other requirements: **MATLAB version 7.0 or newer, R version 2.0 or newer.

• **License**: GNU GPL version 2.

## Abbreviations

OPLS, Orthogonal Projections to Latent Structures; K-OPLS, Kernel-based Orthogonal Projections to Latent Structures; SVM, Support Vector Machine; KPCA, Kernel Principal Component Analysis; KPLS, Kernel Partial Least Squares; NMR, Nuclear Magnetic Resonance; ^1^H HR/MAS NMR, High-resolution magic angle spinning proton NMR; LC-MS, Liquid chromatography-mass spectrometry

## Authors' contributions

MB and MR jointly implemented all provided source code, analysed the *Populus *data set and drafted the manuscript. JKN, EH and JT supervised the project. All authors read and approved the final manuscript.

## Supplementary Material

Additional File 1 Code examples for R and MATLAB. Provides code examples (with illustrations) for running typical tasks using the K-OPLS package for both R and MATLAB Click here for file

Additional File 2 K-OPLS package version 1.0.3 for R (Unix). Provides the K-OPLS package version 1.0.3 for R, built for Unix-like systems (e.g. Linux, MacOS X, etc) Click here for file

Additional File 3 K-OPLS package version 1.0.3 for R (Windows). Provides the K-OPLS package version 1.0.3 for R, built for Windows Click here for file

Additional File 4 K-OPLS package version 1.0.3 for MATLAB. Provides the K-OPLS version 1.0.3 source code and documentation for MATLAB Click here for file
