# Supplementary material for: K-OPLS package: Kernel-based orthogonal projections to latent structures for prediction and interpretation in feature space
Source: BMC Bioinformatics. 2008 Feb 19;9:106. doi: 10.1186/1471-2105-9-106 (PMC2323673; doi:10.1186/1471-2105-9-106)
Supplement: Additional File 1 — Code examples for R and MATLAB. Provides code examples (with illustrations) for running typical tasks using the K-OPLS package for both R and MATLAB [file 1471-2105-9-106-S1.pdf]

Supplementary material to “K-OPLS package: Kernel-based orthogonal projections to latent structures for prediction and interpretation in feature space”

Code examples

All code will be denoted by `this font`. Figures will be shown in the text after the code generating the figure. The illustrated examples are available in a demo in the supplied package. R code is described at pages 1-8 and the respective MATLAB code is described on pages 9-16.

R code example

```
library(kopls)
demo(koplsDemo)

## The data is represented by 1000 spectral variables from two
## different classes. A simple overview is given by singular value
## decomposition (SVD)/ Principal Component Analysis (PCA).
svd.res <- svd(Xtr, nu = 2, nv = 2)
plot(svd.res$u[, 1], svd.res$u[, 2], col = col.vec, pch = pch.vec,
  xlab = "PC1", ylab = "PC2", main = "PCA of original data set")
```

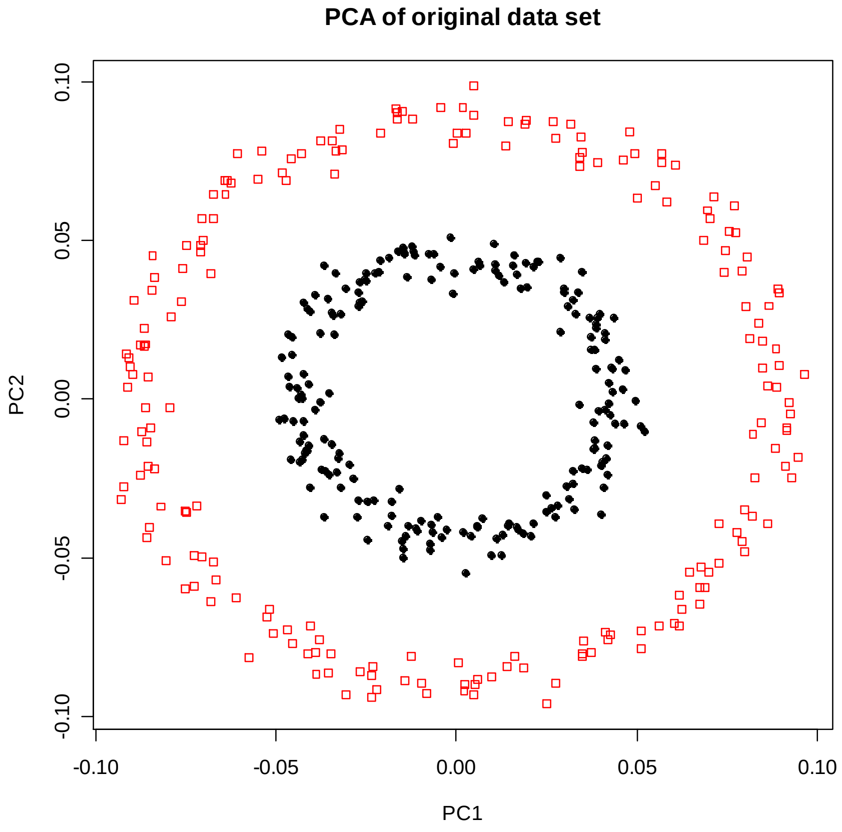

Figure S1. PCA of the original training data.

```
## Define kernel function parameter
sigma<-25

## Construct kernel using the Gaussian kernel function
Ktr<-koplsKernel(Xtr,NULL,'g',sigma)

## Find optimal number of Y-orthogonal components using cross-
## validation
modelCV <-koplsCV(Ktr,Ytr,1,3,nrcv=7,cvType='nfold',
 preProcK='mc',preProcY='mc',modelType='da')

## Visualize results
koplsPlotCVDiagnostics(modelCV)
title("Statistics from K-OPLS cross-validation of original data")
```

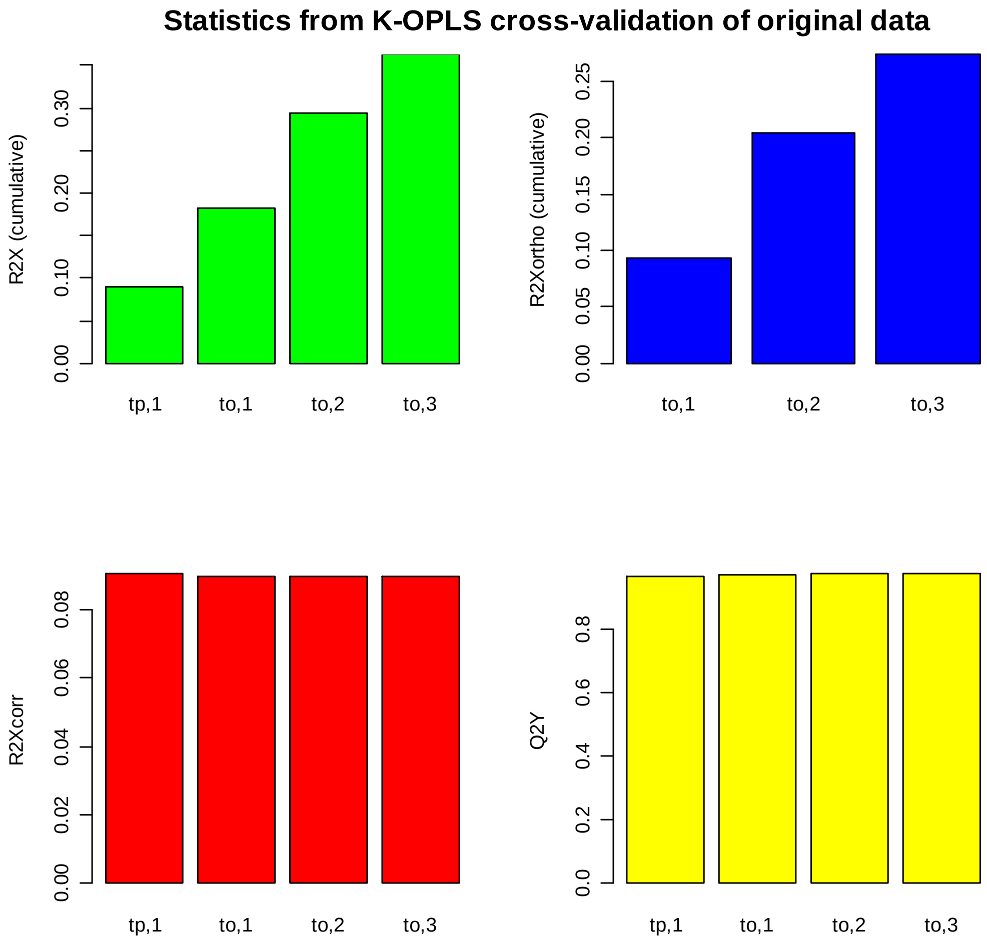

Figure S2. Model statistics of the original data.

```
## We pick nox=1 for diagnostics, although nox=0 is more or less
## equivalent
nox<-1

## Create test kernels for prediction
KteTr<-koplsKernel(Xte,Xtr,'g',sigma)
KteTe<-koplsKernel(Xte,NULL,'g',sigma)

## Create K-OPLS model...
modelOrg<-koplsModel(Ktr,Ytr,1,nox,'mc','mc')

## ... and predict external test set
modelOrgPred<-koplsPredict(KteTr,KteTe,Ktr,modelOrg,rescaleY=TRUE)

## View model scores
koplsPlotScores(modelOrg, col=col.vec, pch=pch.vec)
title("Scatter plot matrix of scores")
```

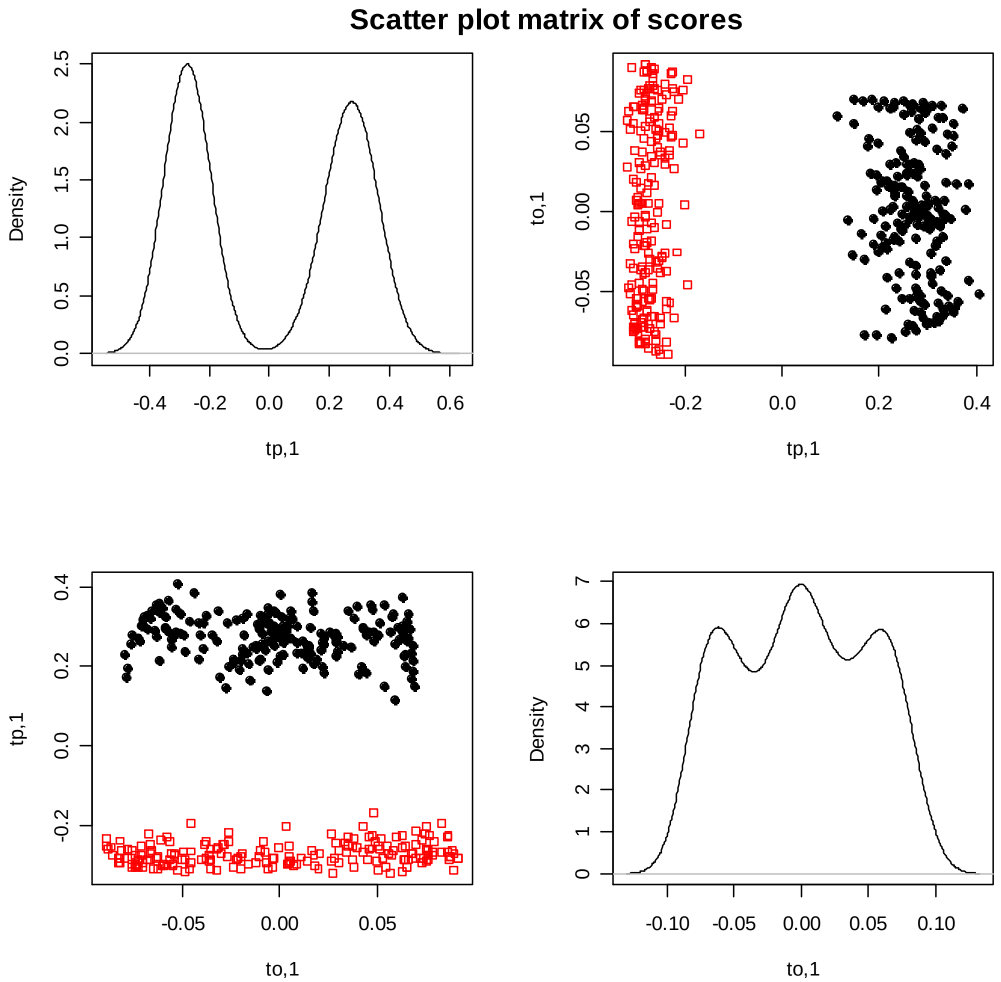

Figure S3. Model scores as scatter plot matrix. Diagonal describes density of that particular model component.

```
## View predictions for external test set
plot(modelOrgPred$Yhat, Yte, xlab="Predicted", ylab="Observed",
 main="Obs. vs. pred. for original data")
abline(v=0.5, lty=2, col="red")
```

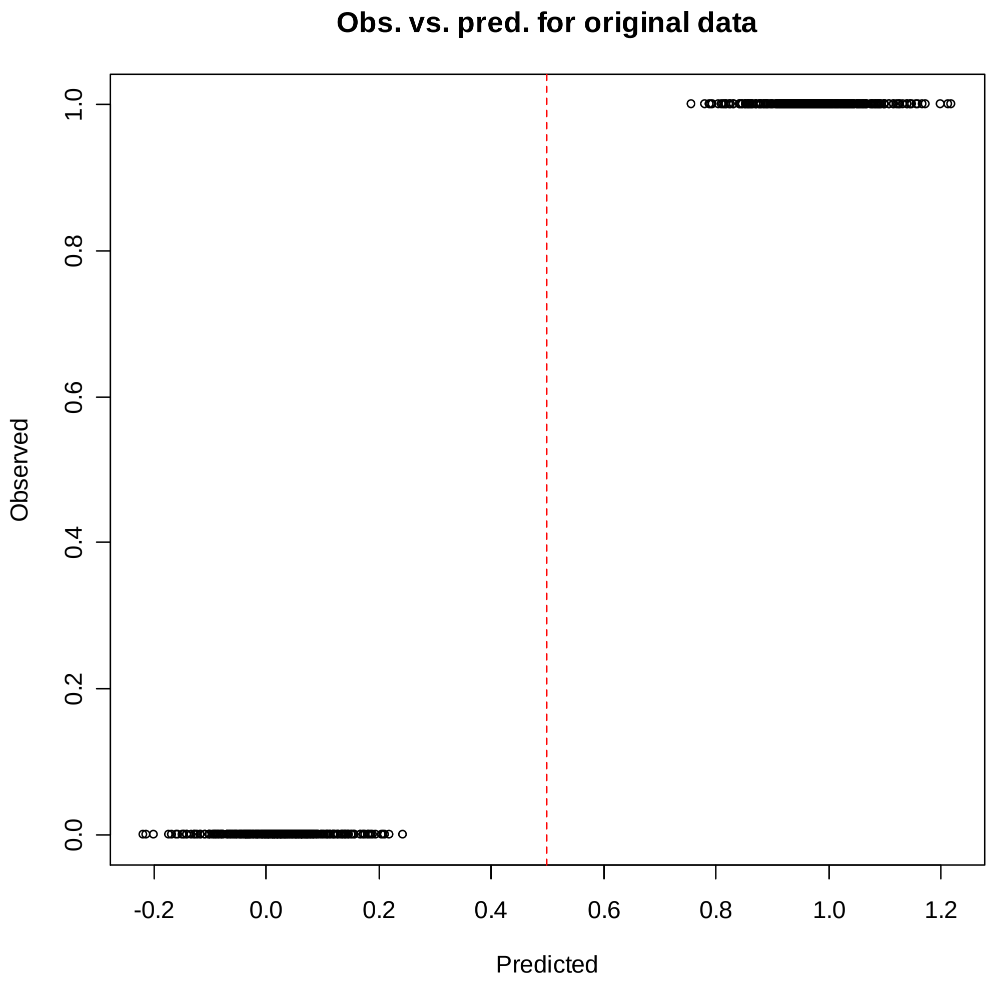

Figure S4. Observed values (0 or 1, denoting class belonging) versus predicted values. The dashed line denotes an approximate decision boundary.

```
## We now model data where class-independent variation is added to
## the original data. This distorts the symmetric circular
## structures, in particular for one of the classes.
svd.reso<-svd(Xtro, nu=2, nv=2)
plot(svd.reso$u[,1], svd.reso$u[,2], col=col.vec, pch=pch.vec,
 xlab="PC1", ylab="PC2",
 main="PCA of data with Y-ortho variation added")
```

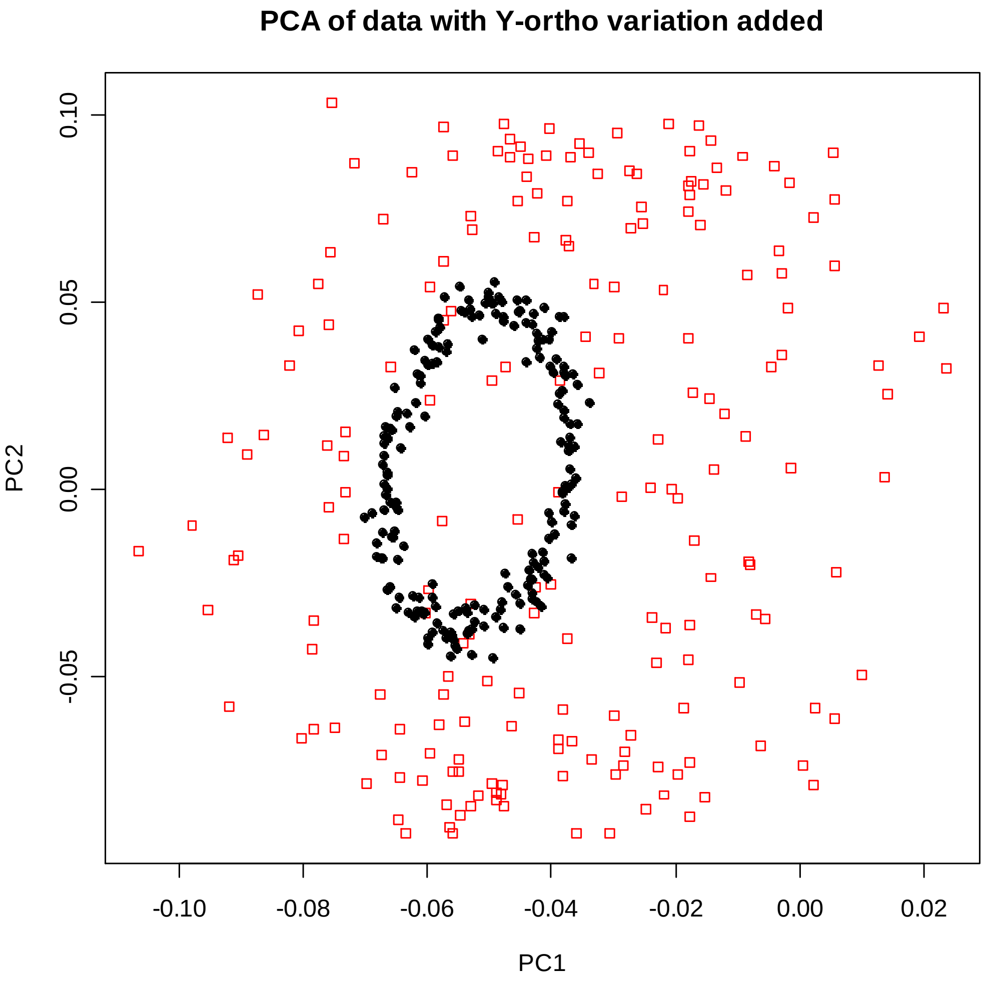

Figure S5. Overview of data set with **Y**-orthogonal variation added, causing one of the classes to become distorted.

```
## create kernels for training and prediction
Ktro<-koplsKernel(Xtro,NULL,'g',sigma)
KteTro<-koplsKernel(Xteo,Xtro,'g',sigma)
KteTeo<-koplsKernel(Xteo,NULL,'g',sigma)

## Model and predict (cross-validation not performed)
modelOSC<-koplsModel(Ktro,Ytr,1,nox,'mc','mc');
modelOSCPred<-koplsPredict(KteTro,KteTeo,Ktro,modelOSC,rescaleY=TRUE)


## We skip cross-validation this time
koplsPlotModelDiagnostics(modelOSC)
title("Model diagnostics of data (Y-ortho variation added)")
```

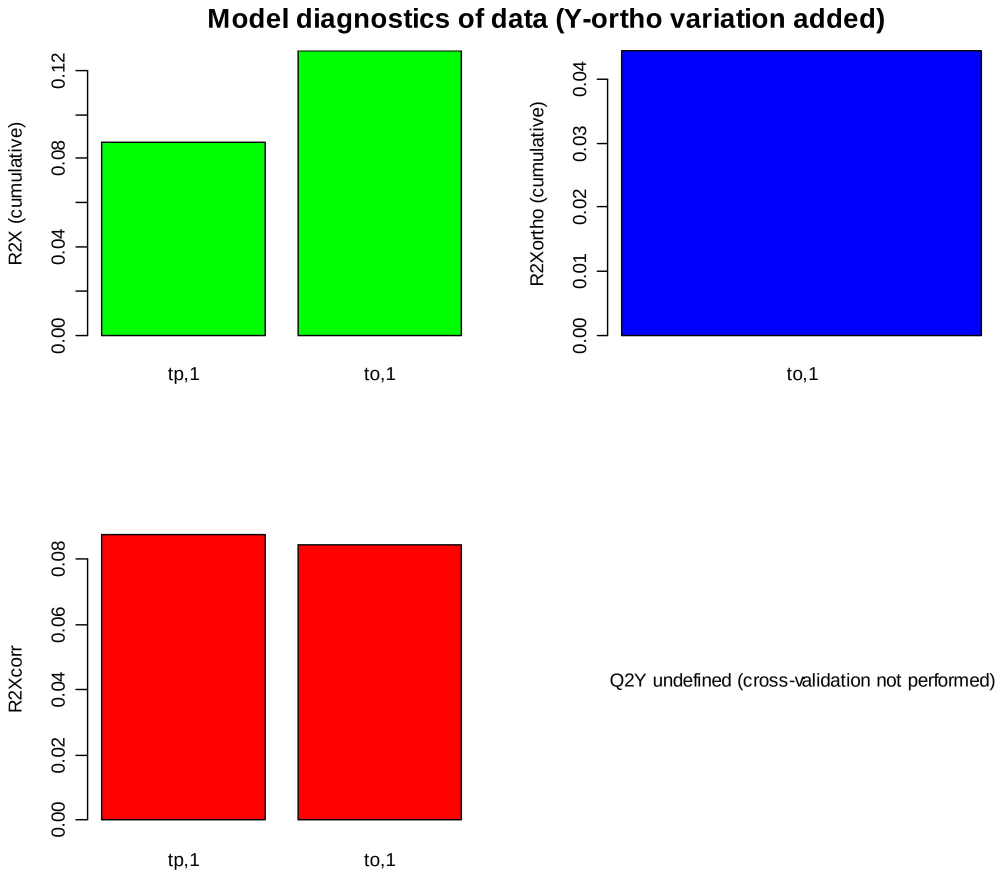

Figure S6. Model diagnostics of data set with **Y**-orthogonal variation added where cross-validation has not been performed.

```
## View scores. Note the magnitude difference between the ‘red’ class
## and the ‘black’ class
koplsPlotScores(modelOSC, col=col.vec, pch=pch.vec)
title("Scatter plot matrix of scores (Y-ortho variation added)")
```

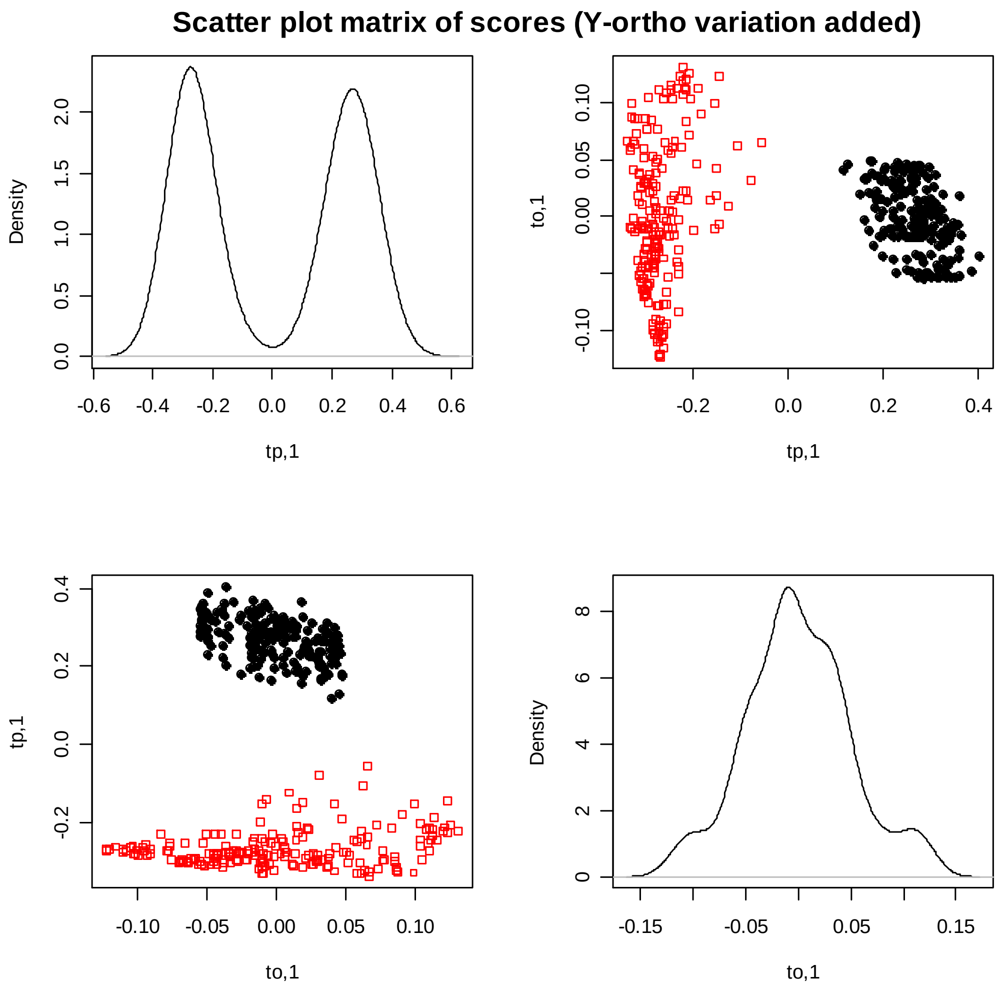

Figure S7. Model scores as a scatter plot matrix for the data set with **Y**-orthogonal variation added. Diagonal describes density of that particular model component.

```
## View predictions for external test set
plot(modelOSCPred$Yhat, Yte, xlab="Predicted", ylab="Observed",
 main="Obs. vs. pred. with Y-ortho variation added")
abline(v=0.5, lty=2, col="red")
```

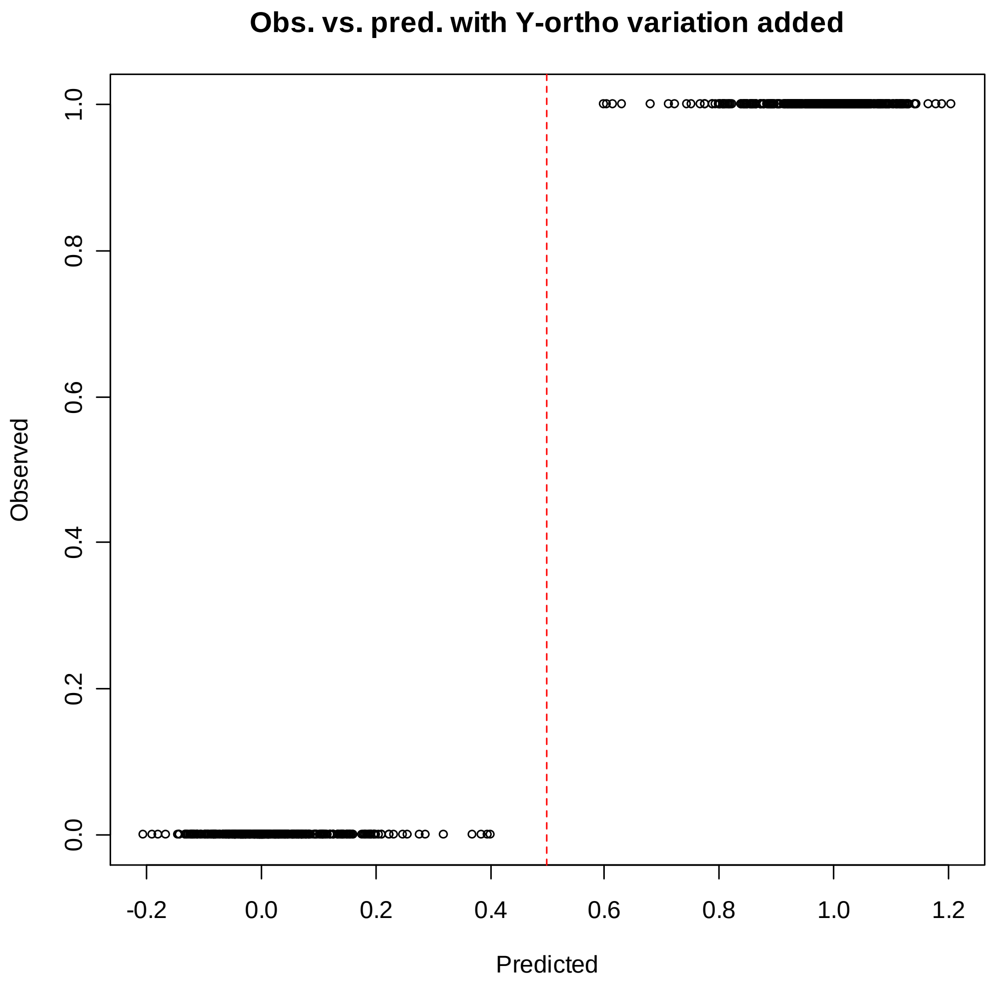

Figure S8. Observed values (0 or 1, denoting class belonging) versus predicted values for the data set with **Y**-orthogonal variation added. The dashed line denotes an approximate decision boundary.

MATLAB code example

```
%% Make sure that all 'kopls*' files are in the current path
%% and that the 'koplsDemo.mat' workspace is loaded

%% The data is represented by 1000 spectral variables from two
%% different classes. A simple overview is given by singular value
%% decomposition (SVD)/ Principal Component Analysis (PCA).
[U,D,V]=svds(Xtr, 2);

%% Overview plot
plot(U(class1,1), U(class1,2), 'bx');
hold on;
plot(U(class2,1), U(class2,2), 'ro');
title('PCA of original data set');
hold off;
```

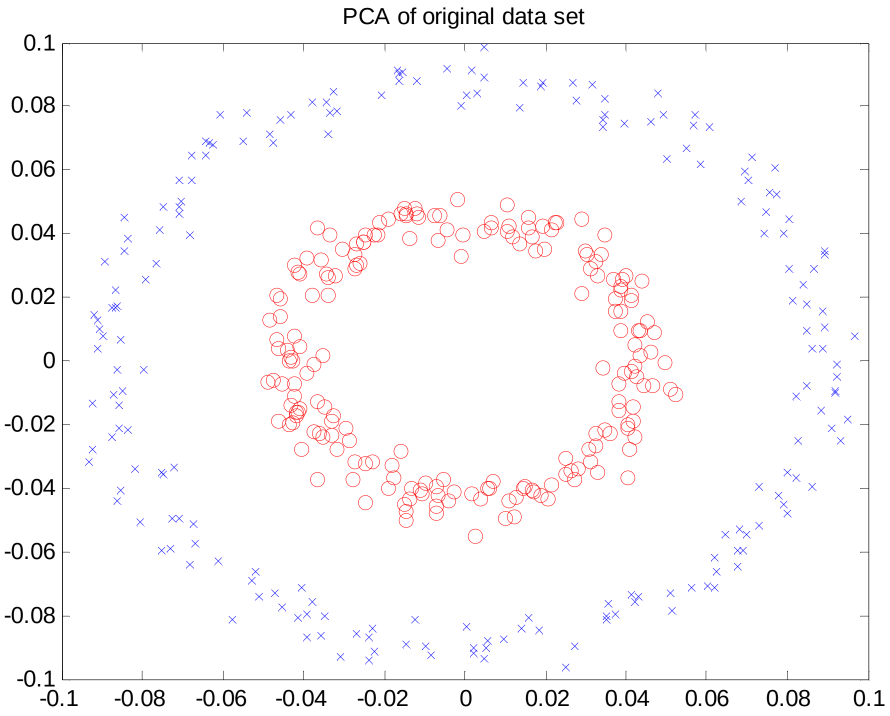

Figure S9. PCA of the original training data.

```
%% Define kernel function parameter
sigma=25;

%% Construct kernel using the Gaussian kernel function
Ktr=koplsKernel(Xtr,[],'g',sigma);

%% Find optimal number of Y-orthogonal components using cross-
%% validation
modelCV=koplsCV(Ktr,Ytr,1,3,7,'nfold','mc','mc',0.75,'da');

%% Visualize results
koplsPlotCVDiagnostics(modelCV);
```

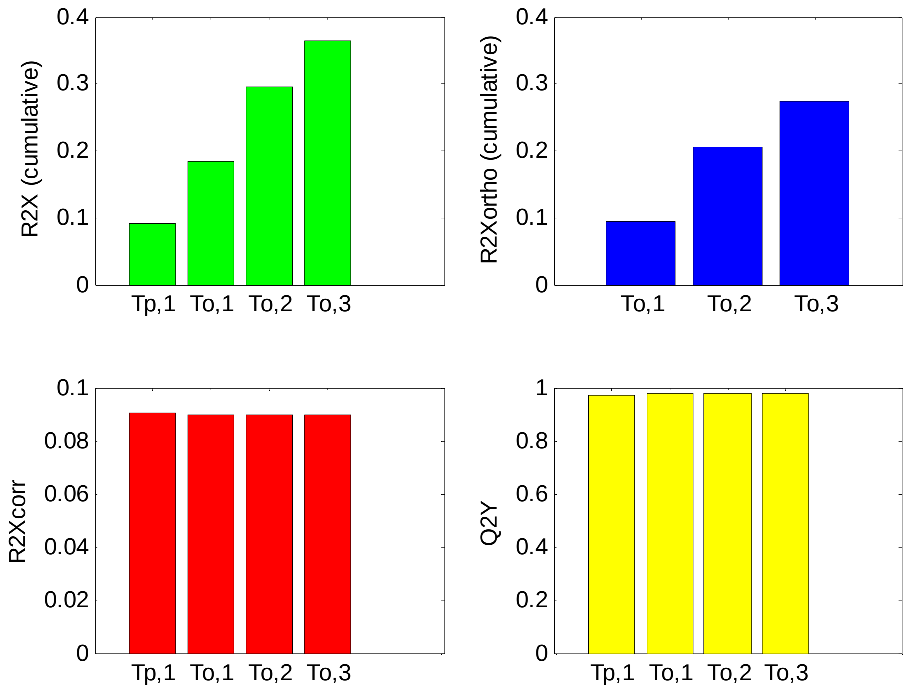

Figure S10. Model statistics of the original data.

```
%% We pick nox=1 for diagnostics, although nox=0 is more or less
%% equivalent
nox=1;

%% Create test kernels for prediction
KteTr=koplsKernel(Xte,Xtr,'g',sigma);
KteTe=koplsKernel(Xte,[],'g',sigma);

%% Create K-OPLS model...
modelOrg=koplsModel(Ktr,Ytr,1,nox,'mc','mc');

%% ... and predict external test set
modelOrgPred=koplsPredict(KteTr,KteTe,Ktr,modelOrg,nox,1);

%% View model scores
koplsPlotScores(modelOrg)
```

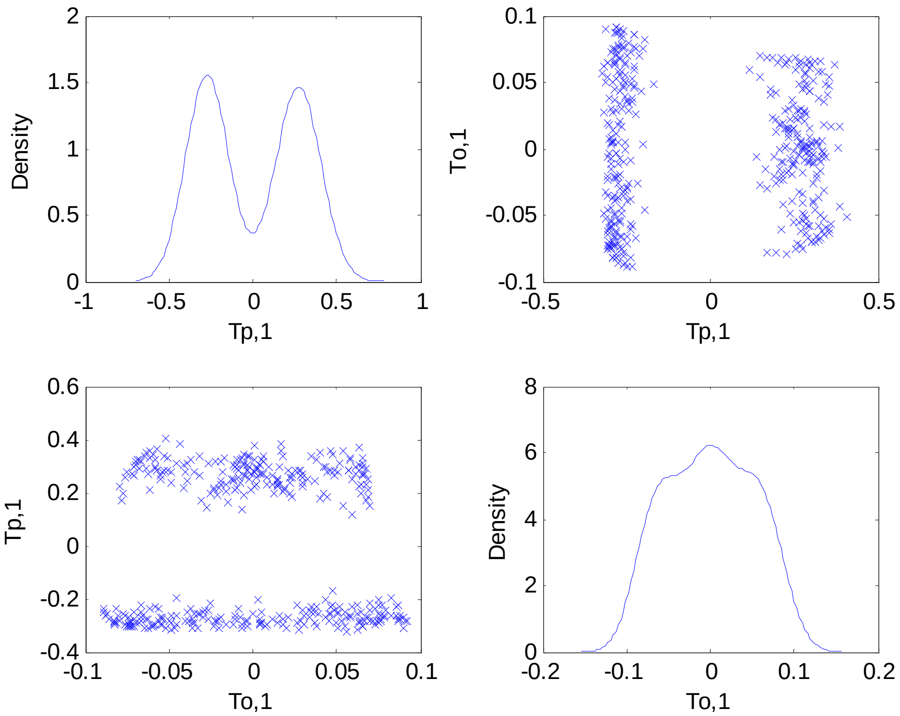

Figure S11. Model scores as scatter plot matrix. Diagonal describes density of that particular model component.

```
%% View predictions for external test set
plot(modelOrgPred.Yhat, Yte, 'xb');
hold on;
xlabel('Predicted');
ylabel('Observed');
title('Obs. vs. pred. for original data');
plot( [0.5 0.5], [0 1], 'r--');
hold off;
```

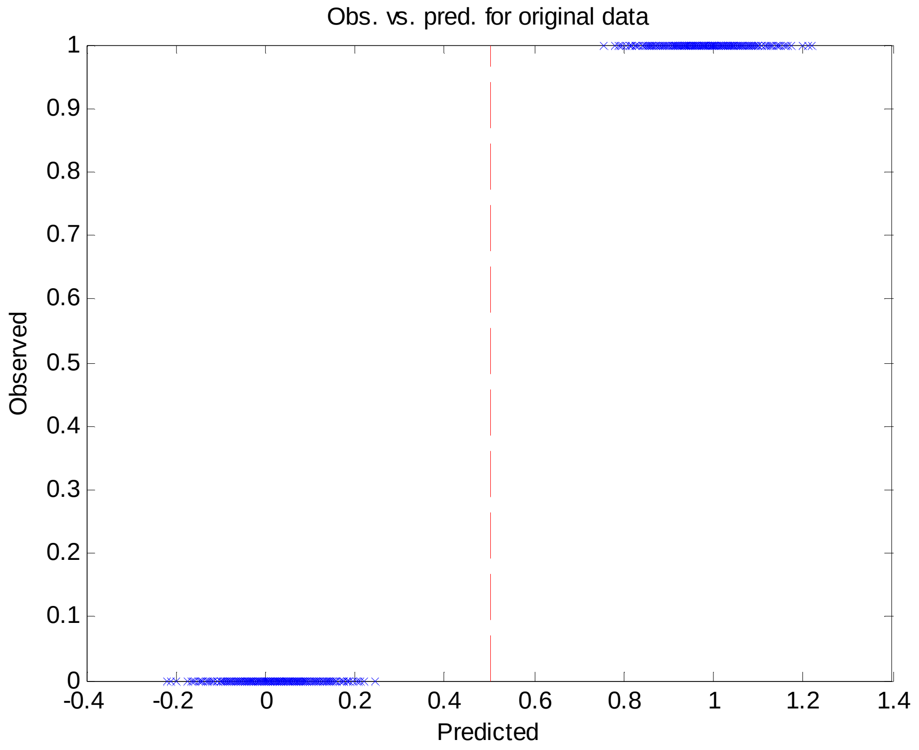

Figure S12. Observed values (0 or 1, denoting class belonging) versus predicted values. The dashed line denotes an approximate decision boundary.

```
%% We now model data where class-independent variation is added to
%% the original data. This distorts the symmetric circular
%% structures, in particular for one of the classes.
[Uo,Do,Vo]=svds(Xtro, 2);

plot(Uo(class1,1), Uo(class1,2), 'bx')
hold on;
plot(Uo(class2,1), Uo(class2,2), 'ro')
title('PCA of data with Y-ortho variation added')
hold off
```

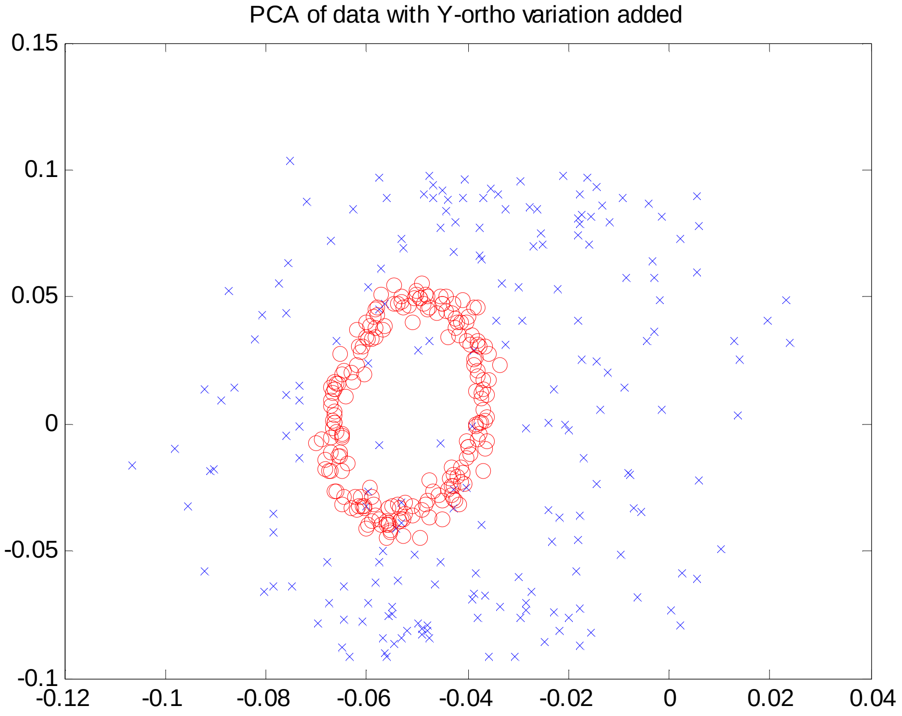

Figure S13 Overview of data set with **Y**-orthogonal variation added, causing one of the classes to become distorted.

```
%% create kernels for training and prediction
Ktro=koplsKernel(Xtro,[],'g',sigma);
KteTro=koplsKernel(Xteo,Xtro,'g',sigma);
KteTeo=koplsKernel(Xteo,[],'g',sigma);

%% Model and predict (cross-validation not performed)
modelOSC=koplsModel(Ktro,Ytr,1,nox,'mc','mc');
modelOSCPred=koplsPredict(KteTro,KteTeo,Ktro,modelOSC,nox,1);


%% We skip cross-validation this time
koplsPlotModelDiagnostics(modelOSC);
```

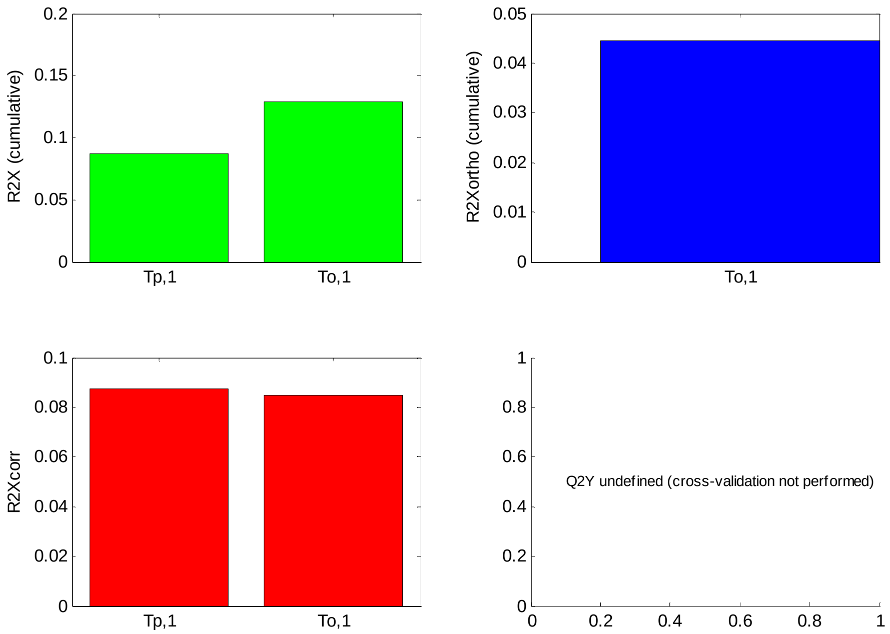

Figure S14. Model diagnostics of data set with **Y**-orthogonal variation added where cross-validation has not been performed.

```
%% View scores. Note the magnitude difference between the classes
koplsPlotScores(modelOSC);
```

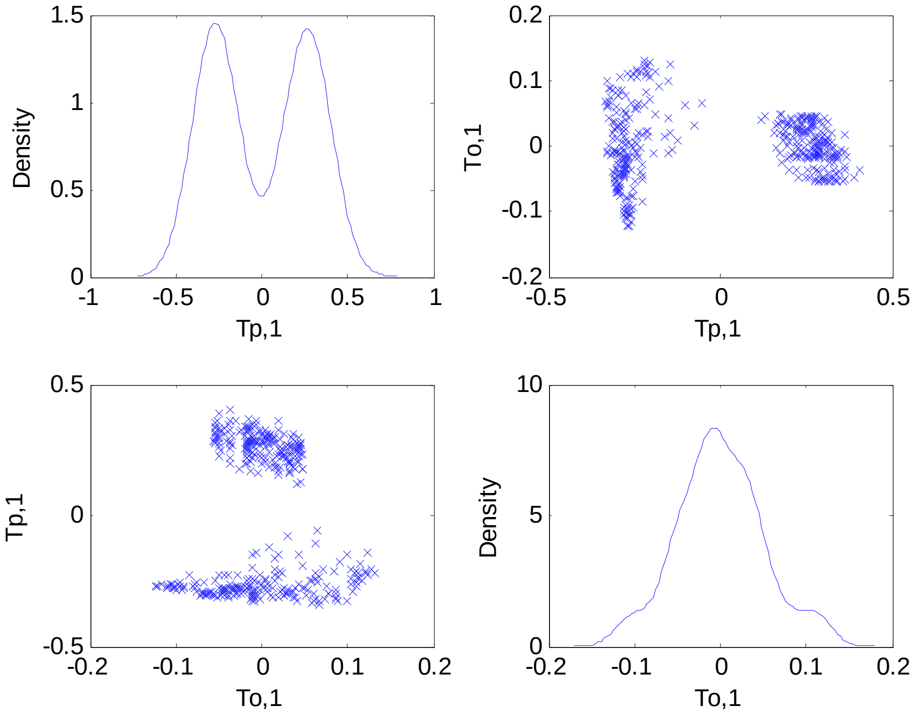

Figure S15. Model scores as a scatter plot matrix for the data set with **Y**-orthogonal variation added. Diagonal describes density of that particular model component.

```
%% View predictions for external test set
plot(modelOSCPred.Yhat, Yte, 'bx');
hold on
xlabel('Predicted');
ylabel('Observed');
title('Obs. vs. pred. with Y-ortho variation added');
plot( [0.5 0.5], [0 1], 'r--');
hold off;
```

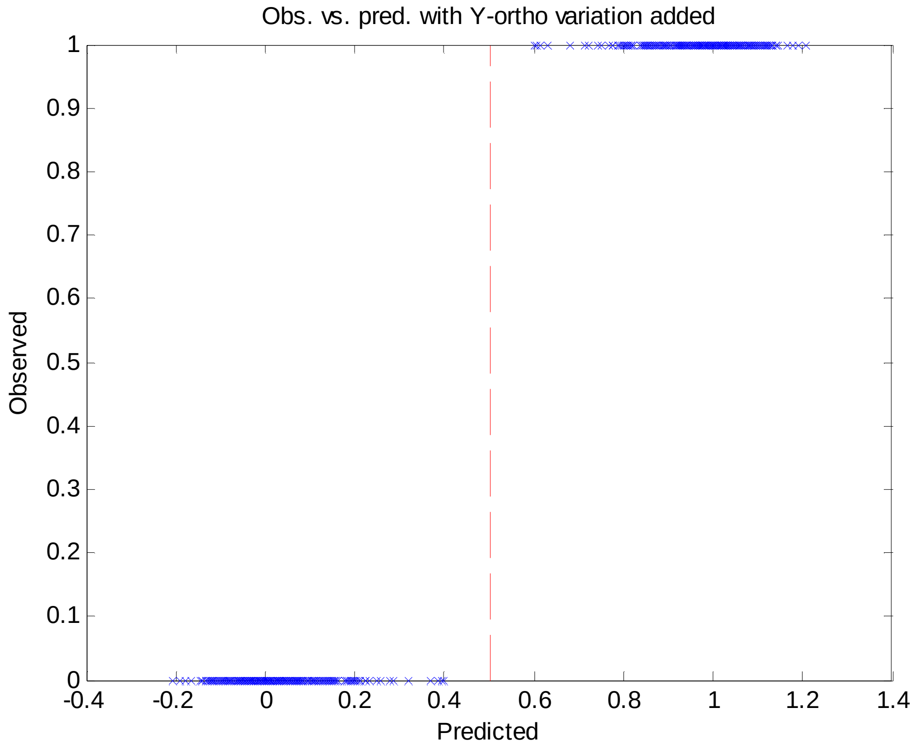

Figure S16. Observed values (0 or 1, denoting class belonging) versus predicted values for the data set with **Y**-orthogonal variation added. The dashed line denotes an approximate decision boundary.
