# Supplementary material for: K-OPLS package: Kernel-based orthogonal projections to latent structures for prediction and interpretation in feature space
Source: BMC Bioinformatics. 2008 Feb 19;9:106. doi: 10.1186/1471-2105-9-106 (PMC2323673; doi:10.1186/1471-2105-9-106)
Supplement: Additional File 3 — K-OPLS package version 1.0.3 for R (Windows). Provides the K-OPLS package version 1.0.3 for R, built for Windows [file 1471-2105-9-106-S3.zip › kopls/doc/index.html]

R: kopls vignettes

## Vignettes of package kopls

kopls.pdf:: kopls - introduction
