# Supplementary material for: K-OPLS package: Kernel-based orthogonal projections to latent structures for prediction and interpretation in feature space
Source: BMC Bioinformatics. 2008 Feb 19;9:106. doi: 10.1186/1471-2105-9-106 (PMC2323673; doi:10.1186/1471-2105-9-106)
Supplement: Additional File 3 — K-OPLS package version 1.0.3 for R (Windows). Provides the K-OPLS package version 1.0.3 for R, built for Windows [file 1471-2105-9-106-S3.zip › kopls/html/00Index.html]

R: Implementation of the Kernel-based Orthogonal Projections to
Latent Structures (K-OPLS) method

# Implementation of the Kernel-based Orthogonal Projections to Latent Structures (K-OPLS) method

---

## Documentation for package `kopls' version 1.0.3

## User Guides and Package Vignettes

Read overview or browse directory.

## Help Pages

|  |  |
| --- | --- |
| kopls-package | Kernel-based orthogonal projections to latent structures (K-OPLS) |
| kopls | Kernel-based orthogonal projections to latent structures (K-OPLS) |
| koplsBasicClassify | Classification rule based on a fixed threshold |
| koplsCenterKTeTe | Centering function for the test kernel |
| koplsCenterKTeTr | Centering function for the hybrid test/training kernel |
| koplsCenterKTrTr | Centering function for the training kernel |
| koplsConfusionMatrix | Calculation of confusion matrix |
| koplsCrossValSet | Generate training/test observations for cross-validation |
| koplsCV | K-OPLS cross-validation |
| koplsDemo | K-OPLS demonstration procedure |
| koplsDummy | Convertion of integer vector to dummy matrix |
| koplsKernel | Kernel construction method |
| koplsMaxClassify | Classification rule based on the maximum class belonging |
| koplsModel | K-OPLS model training |
| koplsPlotCVDiagnostics | Overview plot of cross-validation results |
| koplsPlotModelDiagnostics | Overview of model training results |
| koplsPlotScores | Plots scores from trained K-OPLS models |
| koplsPlotSensSpec | Plots sensitivity and specificity results from cross-validation |
| koplsPredict | Prediction of new samples from a K-OPLS model |
| koplsReDummy | Reconstruct class vector |
| koplsRescale | Matrix scaling based on pre-defined parameters |
| koplsScale | Matrix scaling function |
| koplsScaleApply | Apply matrix scaling |
| koplsSensSpec | Sensitivity and specificity calculations for classification |
