# Supplementary material for: K-OPLS package: Kernel-based orthogonal projections to latent structures for prediction and interpretation in feature space
Source: BMC Bioinformatics. 2008 Feb 19;9:106. doi: 10.1186/1471-2105-9-106 (PMC2323673; doi:10.1186/1471-2105-9-106)
Supplement: Additional File 3 — K-OPLS package version 1.0.3 for R (Windows). Provides the K-OPLS package version 1.0.3 for R, built for Windows [file 1471-2105-9-106-S3.zip › kopls/html/koplsPlotModelDiagnostics.html]

R: Overview of model training results

|  |  |
| --- | --- |
| koplsPlotModelDiagnostics {kopls} | R Documentation |

## Overview of model training results

### Description

Produces overview plots of model training results returned from the `koplsModel` function.

### Usage

```
koplsPlotModelDiagnostics(model, plot.values = FALSE)
```

### Arguments

|  |  |
| --- | --- |
| `model` | The model training result (see `koplsModel` ). |
| `plot.values` | If TRUE, the exact values will be displayed on the bars as text labels. |

### Details

Produces 2x2 panels of bar plots, containing the total explained variation (R2X),
the Y-orthogonal explained variation (R2XO), the Y-correlated explained variation (R2XC)
and (if available) the predicted variation from cross-validation (Q2Y).

### Author(s)

Max Bylesjo and Mattias Rantalainen

### References

Rantalainen M, Bylesjo M, Cloarec O, Nicholson JK, Holmes E and Trygg J.
**Kernel-based orthogonal projections to latent structures (K-OPLS)**, *J Chemometrics* 2007; 21:376-385. doi:10.1002/cem.1071.

### Examples

```
## Load data set
data(koplsExample)

## Define kernel function parameter
sigma<-25

## Define number of Y-orthogonal components
nox<-3

## Construct kernel
Ktr<-koplsKernel(Xtr,NULL,'g',sigma)

## Model 
model<-koplsModel(Ktr,Ytr,1,nox,'mc','mc');

## Visualize results
koplsPlotModelDiagnostics(model)
title("Model diagnostics without cross-validation")
```

---

[Package *kopls* version 1.0.3 Index]
