# Supplementary material for: K-OPLS package: Kernel-based orthogonal projections to latent structures for prediction and interpretation in feature space
Source: BMC Bioinformatics. 2008 Feb 19;9:106. doi: 10.1186/1471-2105-9-106 (PMC2323673; doi:10.1186/1471-2105-9-106)
Supplement: Additional File 3 — K-OPLS package version 1.0.3 for R (Windows). Provides the K-OPLS package version 1.0.3 for R, built for Windows [file 1471-2105-9-106-S3.zip › kopls/html/koplsScale.html]

R: Matrix scaling function

|  |  |
| --- | --- |
| koplsScale {kopls} | R Documentation |

## Matrix scaling function

### Description

Function for mean-centering and scaling of a matrix.

### Usage

```
koplsScale(x, center = "mc", scale = "none")
```

### Arguments

|  |  |
| --- | --- |
| `x` | The matrix to be mean-centered and/or scaled. |
| `center` | Mean-centering type: Either 'mc' for column-wise mean-centering or 'no' for no mean-centering. |
| `scale` | Scaling type: 'uv' for scaling to unit variance, 'pareto' for Pareto scaling (sqrt(uv)) or 'no' for no scaling. |

### Value

A list with the following properties:

|  |  |
| --- | --- |
| `x` | The scaled matrix. |
| `meanVector` | Vector with mean values (possibly) used in the scaling. |
| `sdVector` | Vector with standard deviation values (possibly) used in the scaling. |
| `scale` | Scaling type: see `link{koplsScale}` for details. |
| `center` | Mean-centering type see `link{koplsScale}` for details. |

### Author(s)

Max Bylesjo and Mattias Rantalainen

### References

Rantalainen M, Bylesjo M, Cloarec O, Nicholson JK, Holmes E and Trygg J.
**Kernel-based orthogonal projections to latent structures (K-OPLS)**, *J Chemometrics* 2007; 21:376-385. doi:10.1002/cem.1071.

### Examples

```

```

---

[Package *kopls* version 1.0.3 Index]
