# Supplementary material for: K-OPLS package: Kernel-based orthogonal projections to latent structures for prediction and interpretation in feature space
Source: BMC Bioinformatics. 2008 Feb 19;9:106. doi: 10.1186/1471-2105-9-106 (PMC2323673; doi:10.1186/1471-2105-9-106)
Supplement: Additional File 3 — K-OPLS package version 1.0.3 for R (Windows). Provides the K-OPLS package version 1.0.3 for R, built for Windows [file 1471-2105-9-106-S3.zip › kopls/html/koplsPlotScores.html]

R: Plots scores from trained K-OPLS models

|  |  |
| --- | --- |
| koplsPlotScores {kopls} | R Documentation |

## Plots scores from trained K-OPLS models

### Description

Produces score plots from K-OPLS models. If model components are unspecified,
all possible combinations are displayed as a scatter plot matrix.
Otherwise, two selected components will be shown using a traditional
2D scatter plot.

### Usage

```
koplsPlotScores(model, x = NA, xsub = "p", y = NA, ysub = "o")
```

### Arguments

|  |  |
| --- | --- |
| `model` | K-OPLS model (see `koplsModel`). |
| `x` | x-axis score vector index. |
| `xsub` | Identifying value for `x`: Either 'p' for predictive component or 'o' for Y-orthogonal component. |
| `y` | y-axis score vector index. |
| `ysub` | Identifying value for `y`: Either 'p' for predictive component or 'o' for Y-orthogonal component . |

### Details

The diagnol of the scatter plot matrix depicts the kernel density of that particular score vector.
Any additional parameters will be passed on to the plot() function, which can be used to e.g.
set the color or shape of the displayed data points.

### Author(s)

Max Bylesjo and Mattias Rantalainen

### References

Rantalainen M, Bylesjo M, Cloarec O, Nicholson JK, Holmes E and Trygg J.
**Kernel-based orthogonal projections to latent structures (K-OPLS)**, *J Chemometrics* 2007; 21:376-385. doi:10.1002/cem.1071.

### Examples

```
## Load data set
data(koplsExample)

## Define kernel function parameter
sigma<-25

## Define number of Y-orthogonal components
nox<-3

## Construct kernel
Ktr<-koplsKernel(Xtr,NULL,'g',sigma)

## Model 
model<-koplsModel(Ktr,Ytr,1,nox,'mc','mc');

#### Visualize results

## Shows all scores as scatter plot matrix
## col.vec defines class colors and is loaded by data(koplsExample)
koplsPlotScores(model, col=col.vec)

## Shows tp1 vs to1, colored by class
## pch.vec defines class glyph types and is loaded by data(koplsExample)
koplsPlotScores(model, x=1, xsub='p', y=1, ysub='o', col=col.vec, pch=pch.vec)

## Shows to1 vs to2
koplsPlotScores(model, x=1, xsub='o', y=2, ysub='o', col=col.vec, pch=pch.vec)
```

---

[Package *kopls* version 1.0.3 Index]
