# Supplementary material for: K-OPLS package: Kernel-based orthogonal projections to latent structures for prediction and interpretation in feature space
Source: BMC Bioinformatics. 2008 Feb 19;9:106. doi: 10.1186/1471-2105-9-106 (PMC2323673; doi:10.1186/1471-2105-9-106)
Supplement: Additional File 3 — K-OPLS package version 1.0.3 for R (Windows). Provides the K-OPLS package version 1.0.3 for R, built for Windows [file 1471-2105-9-106-S3.zip › kopls/html/koplsScaleApply.html]

R: Apply matrix scaling

|  |  |
| --- | --- |
| koplsScaleApply {kopls} | R Documentation |

## Apply matrix scaling

### Description

Applies matrix scaling from an external scaling object and returns the scaled matrix with scaling parameters.

### Usage

```
koplsScaleApply(model, x)
```

### Arguments

|  |  |
| --- | --- |
| `model` | Scaled object list, see `link{koplsScale}` for details. |
| `x` | The matrix to be scaled. If NA, the object contained in `model` will be scaled and returned. |

### Value

A list with the following properties:

|  |  |
| --- | --- |
| `x` | The scaled matrix. |
| `meanVector` | Vector with mean values (possibly) used in the scaling. |
| `sdVector` | Vector with standard deviation values (possibly) used in the scaling. |
| `scale` | Scaling type: see `link{koplsScale}` for details. |
| `center` | Mean-centering type see `link{koplsScale}` for details. |

### Author(s)

Max Bylesjo and Mattias Rantalainen

### References

Rantalainen M, Bylesjo M, Cloarec O, Nicholson JK, Holmes E and Trygg J.
**Kernel-based orthogonal projections to latent structures (K-OPLS)**, *J Chemometrics* 2007; 21:376-385. doi:10.1002/cem.1071.

### Examples

```

```

---

[Package *kopls* version 1.0.3 Index]
