# Supplementary material for: K-OPLS package: Kernel-based orthogonal projections to latent structures for prediction and interpretation in feature space
Source: BMC Bioinformatics. 2008 Feb 19;9:106. doi: 10.1186/1471-2105-9-106 (PMC2323673; doi:10.1186/1471-2105-9-106)
Supplement: Additional File 3 — K-OPLS package version 1.0.3 for R (Windows). Provides the K-OPLS package version 1.0.3 for R, built for Windows [file 1471-2105-9-106-S3.zip › kopls/html/koplsDummy.html]

R: Convertion of integer vector to dummy matrix

|  |  |
| --- | --- |
| koplsDummy {kopls} | R Documentation |

## Convertion of integer vector to dummy matrix

### Description

Converts integer vector to binary class matrix ('dummy' matrix).

### Usage

```
koplsDummy(class)
```

### Arguments

|  |  |
| --- | --- |
| `class` | Integer vector containing values denoting class belonging. |
| `numClasses` | Number of classes. If NA, the number of unique entries in `class` will be used instead. |

### Value

A matrix with rows corresponding to observations and columns to
classes. Each element in matrix is either one (observation
belongs to class) or zero (observation does not belong to class).

### Author(s)

Max Bylesjo and Mattias Rantalainen

### References

Rantalainen M, Bylesjo M, Cloarec O, Nicholson JK, Holmes E and Trygg J.
**Kernel-based orthogonal projections to latent structures (K-OPLS)**, *J Chemometrics* 2007; 21:376-385. doi:10.1002/cem.1071.

### Examples

```

```

---

[Package *kopls* version 1.0.3 Index]
