# Supplementary material for: K-OPLS package: Kernel-based orthogonal projections to latent structures for prediction and interpretation in feature space
Source: BMC Bioinformatics. 2008 Feb 19;9:106. doi: 10.1186/1471-2105-9-106 (PMC2323673; doi:10.1186/1471-2105-9-106)
Supplement: Additional File 3 — K-OPLS package version 1.0.3 for R (Windows). Provides the K-OPLS package version 1.0.3 for R, built for Windows [file 1471-2105-9-106-S3.zip › kopls/html/koplsModel.html]

R: K-OPLS model training

|  |  |
| --- | --- |
| koplsModel {kopls} | R Documentation |

## K-OPLS model training

### Description

Function for training a K-OPLS model. The function constructs a predictive regression
model for predicting the values of `Y` by using the information in `K`.
The explained variation is separated into predictive components,
which dimensionality is determined by the parameter `A`, and `Y`-orthogonal components;
dimensionality determined by the parameter `nox`.

### Usage

```
koplsModel(K, Y, A, nox, preProcK = "mc", preProcY = "mc")
```

### Arguments

|  |  |
| --- | --- |
| `K` | Kernel matrix (un-centered); K = <phi(Xtr),phi(Xtr)> |
| `Y` | Response matrix (un-centered/scaled). |
| `A` | Number of predictive components. |
| `nox` | Number of `Y`-orthogonal components. |
| `preProcK` | Pre-processing parameters for the `K` matrix: 'mc' for mean-centering, 'no' for no centering. |
| `preProcY` | Pre-processing parameters for the `Y` matrix: 'mc' for mean-centering, 'uv' for mc + scaling to unit variance, 'pa' for mc + Pareto, 'no' for no scaling. |

### Details

### Value

List with the following components:

|  |  |
| --- | --- |
| `Cp` | `Y` loading matrix. |
| `Sp` | Sigma matrix, containing singular values from `Y`'\*`K`\*`Y` used for scaling. |
| `Sps` | Sp^(-1/2). |
| `Up` | `Y` score matrix. |
| `Tp` | Predictive score matrix for all `Y`-orthogonal components. |
| `T` | Predictive score matrix for the final `Y`-orthogonal component model. |
| `co` | `Y`-orthogonal loading vectors. |
| `so` | Eigenvalues from estimation of `Y`-orthogonal loading vectors. |
| `To` | `Y`-orthogonal score matrix. |
| `toNorm` | Norm of the `Y`-orthogonal score matrix prior to scaling. |
| `Bt` | T-U regression coefficients for predictions. |
| `A` | Number of predictive components. |
| `nox` | Number of `Y`-orthogonal components. |
| `K` | The kernel matrix. |
| `EEprime` | The deflated kernel matrix for residual statistics. |
| `sstot_K` | Total sums of squares in `K` |
| `R2X` | Cumulative explained variation for all model components. |
| `R2XO` | Cumulative explained variation for `Y`-orthogonal model components. |
| `R2XC` | Explained variation for predictive model components after addition of `Y`-orthogonal model components. |
| `sstot_Y` | Total sums of squares in `Y`. |
| `R2Y` | Explained variation of `Y`. |
| `preProc` | Pre-processing parameters: K  Pre-processing setting for `K` = `preProcK`.  Y  Pre-processing setting for `Y` = `preProcY`.  paramsY  Scaling parameters for `Y`. |

### Author(s)

Max Bylesjo and Mattias Rantalainen

### References

Rantalainen M, Bylesjo M, Cloarec O, Nicholson JK, Holmes E and Trygg J.
**Kernel-based orthogonal projections to latent structures (K-OPLS)**, *J Chemometrics* 2007; 21:376-385. doi:10.1002/cem.1071.

### Examples

```
## Load data set
data(koplsExample)

## Define kernel function parameter
sigma<-25

## Define number of Y-orthogonal components
nox<-3

## Construct kernel
Ktr<-koplsKernel(Xtr,NULL,'g',sigma)

## Model 
model<-koplsModel(Ktr,Ytr,1,nox,'mc','mc');

## Visualize results
koplsPlotModelDiagnostics(model)
title("Model diagnostics without cross-validation")
```

---

[Package *kopls* version 1.0.3 Index]
