# Supplementary material for: K-OPLS package: Kernel-based orthogonal projections to latent structures for prediction and interpretation in feature space
Source: BMC Bioinformatics. 2008 Feb 19;9:106. doi: 10.1186/1471-2105-9-106 (PMC2323673; doi:10.1186/1471-2105-9-106)
Supplement: Additional File 3 — K-OPLS package version 1.0.3 for R (Windows). Provides the K-OPLS package version 1.0.3 for R, built for Windows [file 1471-2105-9-106-S3.zip › kopls/html/koplsDemo.html]

R: K-OPLS demonstration procedure

|  |  |
| --- | --- |
| koplsDemo {kopls} | R Documentation |

## K-OPLS demonstration procedure

### Description

This script contains a demonstration of the functionality in the
`kopls` package using a simulated data set.

### Usage

```
demo(koplsDemo)
```

### Details

The data set is represented by 1000 spectral variables from two
different classes and is available in the an attached data set.
The demonstration essentially consists of two main steps.

The first step is to demonstrate how K-OPLS handles the
model evaluation (using cross-validation), model building and
subsequent classification of external data from a non-linear data
set. The second step is to demonstrate how K-OPLS works in the
presence of response-independent (`Y`-orthogonal) variation, using
the same data set but with a strong systematic class-specific
disturbance added.

The `koplsExample` data set contains the following objects:

|  |  |
| --- | --- |
| `Xtr` | The training data matrix, with 400 observations |
|  | and 1000 spectral variables. |
| `Xte` | The test data matrix, with 400 observations |
|  | and 1000 spectral variables. |
| `Xtro` | Same data as 'Xtr', but with class-specific |
|  | systematic noise added. |
| `Xteo` | Same data as 'Xte', but with class-specific |
|  | systematic noise added. |
| `Ytr` | A binary matrix of class assignments for the |
|  | training data. |
| `Yte` | A binary matrix of class assignments for the |
|  | test data. |
| `pch.vec` | A vector with character indices |
|  | (for plotting). |
| `col.vec` | A vector with colors (for plotting). |

### Author(s)

Max Bylesjo and Mattias Rantalainen

### References

Rantalainen M, Bylesjo M, Cloarec O, Nicholson JK, Holmes E and Trygg J.
**Kernel-based orthogonal projections to latent structures (K-OPLS)**, *J Chemometrics* 2007; 21:376-385. doi:10.1002/cem.1071.

---

[Package *kopls* version 1.0.3 Index]
