# Supplementary material for: K-OPLS package: Kernel-based orthogonal projections to latent structures for prediction and interpretation in feature space
Source: BMC Bioinformatics. 2008 Feb 19;9:106. doi: 10.1186/1471-2105-9-106 (PMC2323673; doi:10.1186/1471-2105-9-106)
Supplement: Additional File 3 — K-OPLS package version 1.0.3 for R (Windows). Provides the K-OPLS package version 1.0.3 for R, built for Windows [file 1471-2105-9-106-S3.zip › kopls/html/kopls-package.html]

R: Kernel-based orthogonal projections to latent structures (K-OPLS)

|  |  |
| --- | --- |
| kopls-package {kopls} | R Documentation |

## Kernel-based orthogonal projections to latent structures (K-OPLS)

### Description

An implementation of K-OPLS with e.g. model training, prediction, cross-validation and plot tools.
See individual files for a more in-depth description of the available functionality.

### Usage

```
 Model training: See koplsModel() for details.
 Model predictions: See koplsPredict() for details.
 Cross-validation: See koplsCV() for details.
 Model evaluation: See e.g. koplsPlotScores() or koplsPlotCVDiagnostics() for details.
```

### Details

|  |  |
| --- | --- |
| Package: | kopls |
| Type: | Package |
| Version: | 1.0.3 |
| Date: | 2008-01-23 |
| License: | GPL version 2 |

### Author(s)

Max Bylesjo and Mattias Rantalainen

Maintainer: Max Bylesjo <max.bylesjo@chem.umu.se>

### References

Rantalainen M, Bylesjo M, Cloarec O, Nicholson JK, Holmes E and Trygg J.
**Kernel-based orthogonal projections to latent structures (K-OPLS)**, *J Chemometrics* 2007; 21:376-385. doi:10.1002/cem.1071.

### Examples

```
  ##Run demo to get further info:
  demo(koplsDemo)
```

---

[Package *kopls* version 1.0.3 Index]
