# Supplementary material for: K-OPLS package: Kernel-based orthogonal projections to latent structures for prediction and interpretation in feature space
Source: BMC Bioinformatics. 2008 Feb 19;9:106. doi: 10.1186/1471-2105-9-106 (PMC2323673; doi:10.1186/1471-2105-9-106)
Supplement: Additional File 3 — K-OPLS package version 1.0.3 for R (Windows). Provides the K-OPLS package version 1.0.3 for R, built for Windows [file 1471-2105-9-106-S3.zip › kopls/html/koplsCV.html]

R: K-OPLS cross-validation

|  |  |
| --- | --- |
| koplsCV {kopls} | R Documentation |

## K-OPLS cross-validation

### Description

Function for performing K-OPLS cross-validation for a set of `Y`-orthogonal components.
The function returns a number of diagnostic parameters which can be used to determine the optimal number
of model components.

### Usage

```
koplsCV(K, Y, A, oax, nrcv = 7, cvType = "nfold", preProcK = "mc", preProcY = "mc", cvFrac = 0.75, modelType = "re")
```

### Arguments

|  |  |
| --- | --- |
| `K` | The kernel matrix (un-centered); see `koplsKernel` for details. |
| `Y` | The response matrix (un-centered/scaled). Could be binary (for discriminant analysis) or real-valued. |
| `A` | The number of `Y`-predictive components (integer). |
| `oax` | The number of `Y`-orthogonal components (integer). |
| `nrcv` | Number of cross-validation rounds (integer). |
| `cvType` | Type of cross-validation. Either 'nfold' for n-fold cross-validation, 'mccv' for Monte Carlo CV or 'mccvb' for Monte Carlo class-balanced CV. See also `koplsCrossValSet` for details. |
| `preProcK` | Pre-processing settings for the kernel matrix. Either 'mc' for mean-centering or 'no' for no pre-processing. |
| `preProcY` | Pre-processing parameter for `Y`. Either 'mc' for mean-centering, 'uv' for mc + scaling to unit-variance, 'pareto' for mc + Pareto-scaling or 'no' for no scaling. |
| `cvFrac` | Fraction of observations in the training set during cross-validation. Only applicable for 'mccv' or 'mccvb' cross-validation (see `cvType`) |
| `modelType` | 'da' for discriminant analysis, 're' for regression. If 'da', sensitivity and specificity will be calculated. |

### Details

### Value

|  |  |
| --- | --- |
| `koplsModel` | K-OPLS model object with `A` predictive components and `oax` `Y`-orthogonal components. |
| `cv` | Cross-validation results: Q2Yhat  Total Q-square result for all `Y`-orthogonal components.  Q2YhatVars  Q-square result per `Y`-variable for all `Y`-orthogonal components.  Yhat  All predicted `Y` values as a concatenated matrix.  Tcv  Predictive score vector T for all cross-validation rounds.  cvTrainIndex  Indices for the training set observations during the cross-validation rounds.  cvTestIndex  Indices for the test set observations during the cross-validation rounds. |
| `da` | Cross-validation results specifically for discriminant analysis case: predClass  Predicted class list per class and `Y`-orthogonal components (integer values).  trueClass  Predicted class list per class and `Y`-orthogonal components (integer values).  sensSpec  Sensitivity and specificity values per class and `Y`-orthogonal components (integer values).  confusionMatrix  Confusion matrix during cross-validation rounds.  nclasses  Number of classes in model.  decisionRule  Decision rule used: 'max' or 'fixed'. |
| `args` | Arguments to the function: A  See `A`.  oax  See `oax`. |

### Author(s)

Max Bylesjo and Mattias Rantalainen

### References

Rantalainen M, Bylesjo M, Cloarec O, Nicholson JK, Holmes E and Trygg J.
**Kernel-based orthogonal projections to latent structures (K-OPLS)**, *J Chemometrics* 2007; 21:376-385. doi:10.1002/cem.1071.

### Examples

```
## Load data set
data(koplsExample)

## Define kernel function parameter
sigma<-25 

## Construct kernel
Ktr<-koplsKernel(Xtr,NULL,'g',sigma)

## Find optimal number of Y-orthogonal components by cross-validation
## The cross-validation tests models with Y-orthogonal components 0 through numYo
modelCV<-koplsCV(Ktr,Ytr,1,3,nrcv=7,cvType='nfold',preProcK='mc',preProcY='mc',modelType='da')

## Visualize results
koplsPlotCVDiagnostics(modelCV)
title("Statistics from K-OPLS cross-validation of original data")
```

---

[Package *kopls* version 1.0.3 Index]
