# Supplementary material for: K-OPLS package: Kernel-based orthogonal projections to latent structures for prediction and interpretation in feature space
Source: BMC Bioinformatics. 2008 Feb 19;9:106. doi: 10.1186/1471-2105-9-106 (PMC2323673; doi:10.1186/1471-2105-9-106)
Supplement: Additional File 3 — K-OPLS package version 1.0.3 for R (Windows). Provides the K-OPLS package version 1.0.3 for R, built for Windows [file 1471-2105-9-106-S3.zip › kopls/html/koplsSensSpec.html]

R: Sensitivity and specificity calculations for classification

|  |  |
| --- | --- |
| koplsSensSpec {kopls} | R Documentation |

## Sensitivity and specificity calculations for classification

### Description

Calculates sensitivity and specificity values for classification
in a class-wise fashion.

### Usage

```
koplsSensSpec(trueClass, predClass)
```

### Arguments

|  |  |
| --- | --- |
| `trueClass` | Matrix of true class identifiers (integer). |
| `predClass` | Matrix of predicted class identifiers (integer). |

### Value

A list with the following properties:

|  |  |
| --- | --- |
| `TPtot` | Total true positive (TP) rate. |
| `FPtot` | Total false positive (FP) rate. |
| `TNtot` | Total true negative (TN) rate. |
| `FNtot` | Total false negative (FN) rate. |
| `sensTot` | Overall sensitivity. |
| `specTot` | Overall specificity. |
| `TP` | TP rate for each class. |
| `FP` | FP rate for each class. |
| `TN` | TN rate for each class. |
| `FN` | FN rate for each class. |
| `sens` | Sensitivity for each class. |
| `spec` | Specificity for each class. |
| `Ntot` | Total number of entries. |

### Author(s)

Max Bylesjo and Mattias Rantalainen

### References

Rantalainen M, Bylesjo M, Cloarec O, Nicholson JK, Holmes E and Trygg J.
**Kernel-based orthogonal projections to latent structures (K-OPLS)**, *J Chemometrics* 2007; 21:376-385. doi:10.1002/cem.1071.

### Examples

```

```

---

[Package *kopls* version 1.0.3 Index]
