# Supplementary material for: K-OPLS package: Kernel-based orthogonal projections to latent structures for prediction and interpretation in feature space
Source: BMC Bioinformatics. 2008 Feb 19;9:106. doi: 10.1186/1471-2105-9-106 (PMC2323673; doi:10.1186/1471-2105-9-106)
Supplement: Additional File 3 — K-OPLS package version 1.0.3 for R (Windows). Provides the K-OPLS package version 1.0.3 for R, built for Windows [file 1471-2105-9-106-S3.zip › kopls/html/koplsMaxClassify.html]

R: Classification rule based on the maximum class belonging

|  |  |
| --- | --- |
| koplsMaxClassify {kopls} | R Documentation |

## Classification rule based on the maximum class belonging

### Description

Classification function that determines class belonging based on the maximum value of Yhat
for each class.

### Usage

```
koplsMaxClassify(data)
```

### Arguments

|  |  |
| --- | --- |
| `data` | Matrix of predicted class values (Yhat) for each class. |

### Value

A vector of class belongings (integer values).

### Author(s)

Max Bylesjo and Mattias Rantalainen

### References

Rantalainen M, Bylesjo M, Cloarec O, Nicholson JK, Holmes E and Trygg J.
**Kernel-based orthogonal projections to latent structures (K-OPLS)**, *J Chemometrics* 2007; 21:376-385. doi:10.1002/cem.1071.

### Examples

```

```

---

[Package *kopls* version 1.0.3 Index]
