# Supplementary material for: K-OPLS package: Kernel-based orthogonal projections to latent structures for prediction and interpretation in feature space
Source: BMC Bioinformatics. 2008 Feb 19;9:106. doi: 10.1186/1471-2105-9-106 (PMC2323673; doi:10.1186/1471-2105-9-106)
Supplement: Additional File 3 — K-OPLS package version 1.0.3 for R (Windows). Provides the K-OPLS package version 1.0.3 for R, built for Windows [file 1471-2105-9-106-S3.zip › kopls/html/koplsPredict.html]

R: Prediction of new samples from a K-OPLS model

|  |  |
| --- | --- |
| koplsPredict {kopls} | R Documentation |

## Prediction of new samples from a K-OPLS model

### Description

Performs prediction of new samples from an existing K-OPLS model
(see `koplsModel`).
The function projects the Y-predictive and Y-orthogonal scores components
to predict a value of the response matrix Y.
The dimensionality of the parameters is determined from `model`.

### Usage

```
koplsPredict(KteTr, Ktest, Ktrain, model, nox = NA, rescaleY = FALSE)
```

### Arguments

|  |  |
| --- | --- |
| `KteTr` | The hybrid test/training kernel matrix; KteTr = <phi(Xte),phi(Xtr)>. |
| `Ktest` | The test kernel matrix; Ktest = <phi(Xte),phi(Xte)>. |
| `Ktrain` | The training kernel matrix (same as used in model training); Ktrain = <phi(Xtr),phi(Xtr)> |
| `model` | The trained K-OPLS model (see `koplsModel` ). |
| `nox` | Number of Y-orthogonal score vectors. If undefined, the value used in `model` will be employed. |
| `rescaleY` | If true, the estimated Yhat values will be rescaled according to the scaling parameters in `model`. Otherwise Yhat values will be returned as is (default). |

### Value

|  |  |
| --- | --- |
| `Tp` | Predicted predictive score matrix for all generations 0:`nox` of Y-orthogonal vectors. |
| `T` | Predictive score matrix for the final model with `nox` Y-orthogonal vectors. |
| `to` | Predicted Y-orthogonal score vectors. |
| `EEprime` | Calculated residuals for the test kernel matrix `Ktest`, useful e.g. for residual statistics. |
| `Yhat` | Predicted values of the response matrix. |

### Author(s)

Max Bylesjo and Mattias Rantalainen

### References

Rantalainen M, Bylesjo M, Cloarec O, Nicholson JK, Holmes E and Trygg J.
**Kernel-based orthogonal projections to latent structures (K-OPLS)**, *J Chemometrics* 2007; 21:376-385. doi:10.1002/cem.1071.

### Examples

```
## Load data set
data(koplsExample)

## Define kernel function parameter
sigma<-25

## Define number of Y-orthogonal components
nox<-3

## Construct kernels
Ktr<-koplsKernel(Xtr,NULL,'g',sigma)
KteTr<-koplsKernel(Xte,Xtr,'g',sigma)
KteTe<-koplsKernel(Xte,NULL,'g',sigma)

## Model 
model<-koplsModel(Ktr,Ytr,1,nox,'mc','mc');

## Predict
modelPred<-koplsPredict(KteTr,KteTe,Ktr,model,rescaleY=TRUE)

## Visualize
plot(modelPred$Yhat, Yte, xlab="Predicted", ylab="Observed")
abline(v=0.5, col="Red", lty=2) ## Approximate decision boundary
```

---

[Package *kopls* version 1.0.3 Index]
