# Supplementary material for: K-OPLS package: Kernel-based orthogonal projections to latent structures for prediction and interpretation in feature space
Source: BMC Bioinformatics. 2008 Feb 19;9:106. doi: 10.1186/1471-2105-9-106 (PMC2323673; doi:10.1186/1471-2105-9-106)
Supplement: Additional File 3 — K-OPLS package version 1.0.3 for R (Windows). Provides the K-OPLS package version 1.0.3 for R, built for Windows [file 1471-2105-9-106-S3.zip › kopls/html/koplsKernel.html]

R: Kernel construction method

|  |  |
| --- | --- |
| koplsKernel {kopls} | R Documentation |

## Kernel construction method

### Description

Constructs a kernel matrix K = <phi(`X1`), phi(`X2`)>.
The kernel function k() determines how the data is transformed and
is passed as the separate parameter `Ktype` to the function.
Currently `Ktype` can be either 'g' (Gaussian) or 'p' (polynomial); see the
supplied reference for definitions of these kernel functions.

### Usage

```
koplsKernel(X1, X2, Ktype, param)
```

### Arguments

|  |  |
| --- | --- |
| `X1` | 'Left side' matrix in expression K = <phi(`X1`), phi(`X2`)>. |
| `X2` | 'Right side' matrix in expression K = <phi(`X1`), phi(`X2`)>. |
| `Ktype` | Type of kernel function: either 'g' (Gaussian) or 'p' (polynomial). |
| `param` | A vector with parameters to the kernel function. |

### Details

If the second parameter `X2` is set to NULL, the kernel matrix is considered to be
symmetric and hence the kernel function can be applied at a considerable speed reduction.
This applies generally to pure training kernel or test kernels (where `X1` = `X2`),
but not to a hybrid test/training kernel (where `X1` != `X2`).

### Value

The kernel matrix K, transformed by the kernel function specified by `Ktype`.

### Author(s)

Max Bylesjo and Mattias Rantalainen

### References

Rantalainen M, Bylesjo M, Cloarec O, Nicholson JK, Holmes E and Trygg J.
**Kernel-based orthogonal projections to latent structures (K-OPLS)**, *J Chemometrics* 2007; 21:376-385. doi:10.1002/cem.1071.

### Examples

```
data(koplsExample)

## Define kernel function parameter
sigma<-25

## Construct kernels
Ktr<-koplsKernel(Xtr,NULL,'g',sigma)
KteTr<-koplsKernel(Xte,Xtr,'g',sigma)
KteTe<-koplsKernel(Xte,NULL,'g',sigma)
```

---

[Package *kopls* version 1.0.3 Index]
