# Supplementary material for: K-OPLS package: Kernel-based orthogonal projections to latent structures for prediction and interpretation in feature space
Source: BMC Bioinformatics. 2008 Feb 19;9:106. doi: 10.1186/1471-2105-9-106 (PMC2323673; doi:10.1186/1471-2105-9-106)
Supplement: Additional File 3 — K-OPLS package version 1.0.3 for R (Windows). Provides the K-OPLS package version 1.0.3 for R, built for Windows [file 1471-2105-9-106-S3.zip › kopls/html/koplsCenterKTrTr.html]

R: Centering function for the training kernel

|  |  |
| --- | --- |
| koplsCenterKTrTr {kopls} | R Documentation |

## Centering function for the training kernel

### Description

Centering function for the training kernel, which is constructed
from the training matrix Xtr as K = <phi(Xtr), phi(Xtr)>
(see `koplsKernel` for details on constructing a kernel matrix).

### Usage

```
koplsCenterKTrTr(K)
```

### Arguments

|  |  |
| --- | --- |
| `K` | The kernel matrix; K = <phi(Xtr), phi(Xtr)>. |

### Value

The centered kernel matrix.

### Author(s)

Max Bylesjo and Mattias Rantalainen

### References

Rantalainen M, Bylesjo M, Cloarec O, Nicholson JK, Holmes E and Trygg J.
**Kernel-based orthogonal projections to latent structures (K-OPLS)**, *J Chemometrics* 2007; 21:376-385. doi:10.1002/cem.1071.

### Examples

```
## Load data set
data(koplsExample)

## Define kernel function parameter
sigma<-25

## Construct kernel
Ktr<-koplsKernel(Xtr,NULL,'g',sigma)

## Center kernel
Ktr_centered<-koplsCenterKTrTr(Ktr)
```

---

[Package *kopls* version 1.0.3 Index]
