# Supplementary material for: K-OPLS package: Kernel-based orthogonal projections to latent structures for prediction and interpretation in feature space
Source: BMC Bioinformatics. 2008 Feb 19;9:106. doi: 10.1186/1471-2105-9-106 (PMC2323673; doi:10.1186/1471-2105-9-106)
Supplement: Additional File 3 — K-OPLS package version 1.0.3 for R (Windows). Provides the K-OPLS package version 1.0.3 for R, built for Windows [file 1471-2105-9-106-S3.zip › kopls/html/koplsCenterKTeTr.html]

R: Centering function for the hybrid test/training kernel

|  |  |
| --- | --- |
| koplsCenterKTeTr {kopls} | R Documentation |

## Centering function for the hybrid test/training kernel

### Description

Centering function for the hybrid test/training kernel, which
is constructed from the test matrix Xte and the training matrix
Xtr as KteTr = <phi(Xte), phi(Xtr)>. Requires
additional (un-centered) training kernel to estimate mean values
(see `koplsKernel` for details on constructing a kernel matrix).

### Usage

```
koplsCenterKTeTr(KteTr, Ktrain)
```

### Arguments

|  |  |
| --- | --- |
| `KteTr` | Hybrid test/training kernel matrix; KteTr = <phi(Xte), phi(Xtr)>. |
| `Ktrain` | Training kernel matrix; Ktrain = <phi(Xtr), phi(Xtr)>. |

### Value

The centered test/training kernel matrix.

### Author(s)

Max Bylesjo and Mattias Rantalainen

### References

Rantalainen M, Bylesjo M, Cloarec O, Nicholson JK, Holmes E and Trygg J.
**Kernel-based orthogonal projections to latent structures (K-OPLS)**, *J Chemometrics* 2007; 21:376-385. doi:10.1002/cem.1071.

### Examples

```
## Load data set
data(koplsExample)

## Define kernel function parameter
sigma<-25

## Construct kernels
Ktr<-koplsKernel(Xtr,NULL,'g',sigma)
KteTr<-koplsKernel(Xte,Xtr,'g',sigma)

## Center kernel
KteTr_centered<-koplsCenterKTeTr(KteTr, Ktr)
```

---

[Package *kopls* version 1.0.3 Index]
