# Supplementary material for: K-OPLS package: Kernel-based orthogonal projections to latent structures for prediction and interpretation in feature space
Source: BMC Bioinformatics. 2008 Feb 19;9:106. doi: 10.1186/1471-2105-9-106 (PMC2323673; doi:10.1186/1471-2105-9-106)
Supplement: Additional File 3 — K-OPLS package version 1.0.3 for R (Windows). Provides the K-OPLS package version 1.0.3 for R, built for Windows [file 1471-2105-9-106-S3.zip › kopls/html/koplsCrossValSet.html]

R: Generate training/test observations for cross-validation

|  |  |
| --- | --- |
| koplsCrossValSet {kopls} | R Documentation |

## Generate training/test observations for cross-validation

### Description

Generates sets of training/test observations useful for cross-validation (CV).
How the sets are generated is determined by the `type` parameter,
which can be either 'nfold' for n-fold cross-validation, 'mccv' for Monte Carlo CV,
'mccvb' for Monte Carlo class-balanced CV.

### Usage

```
koplsCrossValSet(K, Y, type = "nfold", nfold, i, trainFrac = (2/3))
```

### Arguments

|  |  |
| --- | --- |
| `K` | Kernel matrix. |
| `Y` | Response matrix. |
| `type` | Type of cross-validation: 'nfold' for n-fold, 'mccv' for Monte Carlo CV, 'mccvb' for Monte Carlo class-balanced CV. |
| `nfold` | Number of total nfold rounds (if type='nfold'). |
| `i` | Current nfold round (if type='nfold'). |
| `trainFrac` | Fraction of observations in training set. |

### Details

If `type` is set to 'nfold', the parameter `nfold` determines the number of rounds,
which are later subindexed by the `i` parameter.
If 'mccv' or 'mccvb', the `trainFrac` parameter determines the fraction of observations
that will belong to the training set; remaining observations end up in the test set.

### Value

List object with the following entries:

|  |  |
| --- | --- |
| `KTrTr` | Kernel training matrix; KTrTr = <phi(Xtr),phi(Xtr)>. |
| `KTeTr` | Kernel test/training matrix; KTeTr = <phi(Xte),phi(Xtr)>. |
| `KTeTe` | Kernel test matrix; KTeTe = <phi(Xte),phi(Xte)>. |
| `yTrain` | Y training set. |
| `yTest` | Y test set. |
| `trainInd` | Indices of training set observations. |
| `testInd` | Indices of test set observations. |

### Author(s)

Max Bylesjo and Mattias Rantalainen

### References

Rantalainen M, Bylesjo M, Cloarec O, Nicholson JK, Holmes E and Trygg J.
**Kernel-based orthogonal projections to latent structures (K-OPLS)**, *J Chemometrics* 2007; 21:376-385. doi:10.1002/cem.1071.

### Examples

```

```

---

[Package *kopls* version 1.0.3 Index]
