# Supplementary material for: K-OPLS package: Kernel-based orthogonal projections to latent structures for prediction and interpretation in feature space
Source: BMC Bioinformatics. 2008 Feb 19;9:106. doi: 10.1186/1471-2105-9-106 (PMC2323673; doi:10.1186/1471-2105-9-106)
Supplement: Additional File 3 — K-OPLS package version 1.0.3 for R (Windows). Provides the K-OPLS package version 1.0.3 for R, built for Windows [file 1471-2105-9-106-S3.zip › kopls/html/koplsPlotSensSpec.html]

R: Plots sensitivity and specificity results from cross-validation

|  |  |
| --- | --- |
| koplsPlotSensSpec {kopls} | R Documentation |

## Plots sensitivity and specificity results from cross-validation

### Description

Plots sensitivity and specificity results from cross-validation
in a bar plot. The produced bars are shown separately for each class
including overall sensitivity and specificity results.

### Usage

```
koplsPlotSensSpec(modelFull)
```

### Arguments

|  |  |
| --- | --- |
| `modelFull` | The 'koplscv' model from cross-validation (see `koplsCV`). |

### Value

The resulting sensitivity and specificity measures.

### Author(s)

Max Bylesjo and Mattias Rantalainen

### References

Rantalainen M, Bylesjo M, Cloarec O, Nicholson JK, Holmes E and Trygg J.
**Kernel-based orthogonal projections to latent structures (K-OPLS)**, *J Chemometrics* 2007; 21:376-385. doi:10.1002/cem.1071.

### Examples

```
## Load data set
data(koplsExample)

## Define kernel function parameter
sigma<-25 

## Construct kernel
Ktr<-koplsKernel(Xtr,NULL,'g',sigma)

## Find optimal number of Y-orthogonal components by cross-validation
## The cross-validation tests models with Y-orthogonal components 0 through numYo
modelCV<-koplsCV(Ktr,Ytr,1,3,nrcv=7,cvType='nfold',preProcK='mc',preProcY='mc',modelType='da')

## Visualize results
koplsPlotSensSpec(modelCV)
```

---

[Package *kopls* version 1.0.3 Index]
