# Supplementary material for: K-OPLS package: Kernel-based orthogonal projections to latent structures for prediction and interpretation in feature space
Source: BMC Bioinformatics. 2008 Feb 19;9:106. doi: 10.1186/1471-2105-9-106 (PMC2323673; doi:10.1186/1471-2105-9-106)
Supplement: Additional File 3 — K-OPLS package version 1.0.3 for R (Windows). Provides the K-OPLS package version 1.0.3 for R, built for Windows [file 1471-2105-9-106-S3.zip › kopls/doc/kopls.pdf]

Kernel-based Orthogonal Projections to Latent Structures (K-OPLS)

Max Bylesjo and Mattias Rantalainen

January 23, 2008

Abstract

This document describes features of the `kopls` package for R. The presented package provides an open-source, platform-independent implementation of the Kernel-based Orthogonal Projections to Latent Structures (K-OPLS) method; a kernel-based classification and regression method. In relation to other kernel-based methods, K-OPLS offers unique properties facilitating separate modeling of predictive variation and structured noise in the feature space. While providing prediction results similar to other kernel-based methods, K-OPLS features enhanced interpretational capabilities; allowing detection of unanticipated systematic variation in the data such as instrumental drift, batch variability or unexpected biological variation.

Contents

1 Features **3**

2 Requirements **3**

3 Installation **3**
3.1 Windows 3
3.2 Linux 3

4 Getting started **4**

5 Availability **4**

6 Specification of functions **4**
koplsBasicClassify 4
koplsCV 5
koplsCenterKTeTe 7
koplsCenterKTeTr 8
koplsCenterKTrTr 9
koplsConfusionMatrix 10
koplsCrossValSet 10
koplsDemo 12
koplsDummy 13
koplsKernel 13
koplsMaxClassify 14
koplsModel 15
koplsPlotCVDiagnostics 17
koplsPlotModelDiagnostics 18
koplsPlotScores 19
koplsPlotSensSpec 20
koplsPredict 21
koplsReDummy 23
koplsRescale 24
koplsScale 24
koplsScaleApply 25
koplsSensSpec 26

1 Features

The package features the following main functionality:

1. Estimation (training) of K-OPLS models.
2. Prediction of new data using the estimated K-OPLS model in the preceding step.
3. Cross-validation functionality to estimate the generalization error of a K-OPLS model. This is intended to guide the selection of the number of Y-predictive components A and the number of Y-orthogonal components A_o. The supported implementations are:
   - n-fold cross-validation.
   - Monte Carlo Cross-Validation (MCCV)
   - Monte Carlo Class-balanced Cross-Validation (for discriminant analysis cases).
4. Kernel functions, including the polynomial and Gaussian kernel functions.
5. Model statistics:
   - The explained variation of X (R^2_X).
   - The explained variation of Y (R^2_Y).
   - Prediction statistics over cross-validation for regression tasks (Q^2_Y, which is inversely proportional to the generalization error).
   - Prediction statistics over cross-validation for classification tasks (sensitivity and specificity measures).
6. Plot functions for visualization:
   - Scatter plot matrices for model score components.
   - Model statistics and diagnostics plots.

2 Requirements

A functional installation of R 2.0 or later is required.

3 Installation

3.1 Windows

Download the `kopls.1.XX.zip` file (where `XX` is the current version). Start R and select (on the menu bar) "Packages" and then "Install package from local zip file..." Locate the file `kopls.1.XX.zip` on your hard drive, and click "Open".

3.2 Linux

To install the kopls package in the standard location (`/usr/local/lib/R/library`), type:

```
R CMD INSTALL kopls.1.XX.tar.gz
```

(where `XX` is the current version)

4 Getting started

The package includes a demonstration of the functionality available in the package, which is intended to be a starting point for future use. The demonstration is based on a simulated data set, represented by 1000 spectral variables from two different classes and is available in a supplied workspace. The demonstration essentially consists two main steps.

The first step is to demonstrate how K-OPLS handles the model evaluation (using cross-validation), model building and subsequent classification of external data from a non-linear data set.

The second step is to demonstrate how K-OPLS works in the presence of response-independent (Y-orthogonal) variation, using the same data set but with a strong systematic class-specific disturbance added.

To start the demonstration, start R and run the following commands:

```
> library(kopls)
> demo(koplsDemo)
```

5 Availability

The K-OPLS package for R is freely available for download at
`http://kopls.sourceforge.net/`.

6 Specification of functions

`koplsBasicClassify` *Classification rule based on a fixed threshold*

Description

Classification function that determines class belonging based on a fixed threshold rule.

Usage

```
koplsBasicClassify(data, k)
```

Arguments

`data`	The predicted values (Yhat)
`k`	The pre-defined threshold

Value

The predicted classes (integer value).

Author(s)

Max Bylesjo and Mattias Rantalainen

References

Rantalainen M, Bylesjo M, Cloarec O, Nicholson JK, Holmes E and Trygg J. **Kernel-based orthogonal projections to latent structures (K-OPLS)**, *J Chemometrics* 2007; 21:376-385. doi:10.1002/cem.1071.

Examples

`koplsCV` *K-OPLS cross-validation*

Description

Function for performing K-OPLS cross-validation for a set of `Y`-orthogonal components. The function returns a number of diagnostic parameters which can be used to determine the optimal number of model components.

Usage

```
koplsCV(K, Y, A, oax, nrcv = 7, cvType = "nfold", preProcK = "mc",
 preProcY = "mc", cvFrac = 0.75, modelType = "re")
```

Arguments

`K`	The kernel matrix (un-centered); see `koplsKernel` for details.
`Y`	The response matrix (un-centered/scaled). Could be binary (for discriminant analysis) or real-valued.
`A`	The number of `Y`-predictive components (integer).
`oax`	The number of `Y`-orthogonal components (integer).
`nrcv`	Number of cross-validation rounds (integer). ,
`cvType`	Type of cross-validation. Either 'nfold' for n-fold cross-validation, 'mccv' for Monte Carlo CV or 'mccvb' for Monte Carlo class-balanced CV. See also `koplsCrossValSet` for details.
`preProcK`	Pre-processing settings for the kernel matrix. Either 'mc' for mean-centering or 'no' for no pre-processing. ,
`preProcY`	Pre-processing parameter for `Y`. Either 'mc' for mean-centering, 'uv' for mc + scaling to unit-variance, 'pareto' for mc + Pareto-scaling or 'no' for no scaling.
`cvFrac`	Fraction of observations in the training set during cross-validation. Only applicable for 'mccv' or 'mccvb' cross-validation (see `cvType`)
`modelType`	'da' for discriminant analysis, 're' for regression. If 'da', sensitivity and specificity will be calculated.

Details

Value

`koplsModel` K-OPLS model object with `A` predictive components and `oax` `Y`-orthogonal components.

`cv` Cross-validation results:

Q2Yhat Total Q-square result for all `Y`-orthogonal components.

Q2YhatVars Q-square result per `Y`-variable for all `Y`-orthogonal components.

Yhat All predicted `Y` values as a concatenated matrix.

Tcv Predictive score vector T for all cross-validation rounds.

cvTrainIndex Indices for the training set observations during the cross-validation rounds.

cvTestIndex Indices for the test set observations during the cross-validation rounds.

`da` Cross-validation results specifically for discriminant analysis case:

predClass Predicted class list per class and `Y`-orthogonal components (integer values).

trueClass Predicted class list per class and `Y`-orthogonal components (integer values).

sensSpec Sensitivity and specificity values per class and `Y`-orthogonal components (integer values).

confusionMatrix Confusion matrix during cross-validation rounds.

nclasses Number of classes in model.

decisionRule Decision rule used: 'max' or 'fixed'.

`args` Arguments to the function:

A See `A`.

oax See `oax`.

Author(s)

Max Bylesjo and Mattias Rantalainen

References

Rantalainen M, Bylesjo M, Cloarec O, Nicholson JK, Holmes E and Trygg J. **Kernel-based orthogonal projections to latent structures (K-OPLS)**, *J Chemometrics* 2007; 21:376-385. doi:10.1002/cem.1071.

Examples

```
## Load data set
data(koplsExample)

## Define kernel function parameter
sigma<-25

## Construct kernel
Ktr<-koplsKernel(Xtr,NULL,'g',sigma)

## Find optimal number of Y-orthogonal components by cross-validation
## The cross-validation tests models with Y-orthogonal components 0
through numYo
```

```
modelCV<-koplsCV(Ktr,Ytr,1,3,nrcv=7,cvType='nfold',preProcK='mc',
preProcY='mc',modelType='da')

## Visualize results
koplsPlotCVDiagnostics(modelCV)
title("Statistics from K-OPLS cross-validation of original data")
```

`koplsCenterKTeTe` *Centering function for the test kernel*

Description

Centering function for the test kernel, which is constructed from the test matrix Xte as KteTe = <phi(Xte), phi(Xte)>. Requires additional (un-centered) kernels KteTr and KteTr to estimate mean values (see `koplsKernel` for details on constructing a kernel matrix).

Usage

```
koplsCenterKTeTe(KteTe, KteTr, KtrTr)
```

Arguments

`KteTe`	Test kernel matrix; KteTe = <phi(Xte), phi(Xte)>.
`KteTr`	Test/training kernel matrix; KteTr = <phi(Xte), phi(Xtr)>.
`KtrTr`	Training kernel matrix; KtrTr = <phi(Xtr), phi(Xtr)>.

Value

The centered test kernel matrix.

Author(s)

Max Bylesjo and Mattias Rantalainen

References

Rantalainen M, Bylesjo M, Cloarec O, Nicholson JK, Holmes E and Trygg J. **Kernel-based orthogonal projections to latent structures (K-OPLS)**, *J Chemometrics* 2007; 21:376-385. doi:10.1002/cem.1071.

Examples

```
## Load data set
data(koplsExample)

## Define kernel function parameter
sigma<-25

## Construct kernels
Ktr<-koplsKernel(Xtr,NULL,'g',sigma)
KteTr<-koplsKernel(Xte,Xtr,'g',sigma)
```

```
KteTe<-koplsKernel(Xte,NULL,'g',sigma)

## Center kernel
KteTe_centered<-koplsCenterKTeTe(KteTe, KteTr, Ktr)
```

`koplsCenterKTeTr` *Centering function for the hybrid test/training kernel*

Description

Centering function for the hybrid test/training kernel, which is constructed from the test matrix Xte and the training matrix Xtr as KteTr = <phi(Xte), phi(Xtr)>. Requires additional (un-centered) training kernel to estimate mean values (see `koplsKernel` for details on constructing a kernel matrix).

Usage

```
koplsCenterKTeTr(KteTr, Ktrain)
```

Arguments

`KteTr`	Hybrid test/training kernel matrix; KteTr = <phi(Xte), phi(Xtr)>.
`Ktrain`	Training kernel matrix; Ktrain = <phi(Xtr), phi(Xtr)>.

Value

The centered test/training kernel matrix.

Author(s)

Max Bylesjo and Mattias Rantalainen

References

Rantalainen M, Bylesjo M, Cloarec O, Nicholson JK, Holmes E and Trygg J. **Kernel-based orthogonal projections to latent structures (K-OPLS)**, *J Chemometrics* 2007; 21:376-385. doi:10.1002/cem.1071.

Examples

```
## Load data set
data(koplsExample)

## Define kernel function parameter
sigma<-25

## Construct kernels
Ktr<-koplsKernel(Xtr,NULL,'g',sigma)
KteTr<-koplsKernel(Xte,Xtr,'g',sigma)

## Center kernel
KteTr_centered<-koplsCenterKTeTr(KteTr, Ktr)
```

`koplsCenterKTrTr` *Centering function for the training kernel*

Description

Centering function for the training kernel, which is constructed from the training matrix Xtr as K = <phi(Xtr), phi(Xtr)> (see `koplsKernel` for details on constructing a kernel matrix).

Usage

```
koplsCenterKTrTr(K)
```

Arguments

`K`	The kernel matrix; K = <phi(Xtr), phi(Xtr)>.

Value

The centered kernel matrix.

Author(s)

Max Bylesjo and Mattias Rantalainen

References

Rantalainen M, Bylesjo M, Cloarec O, Nicholson JK, Holmes E and Trygg J. **Kernel-based orthogonal projections to latent structures (K-OPLS)**, *J Chemometrics* 2007; 21:376-385. doi:10.1002/cem.1071.

Examples

```
## Load data set
data(koplsExample)

## Define kernel function parameter
sigma<-25

## Construct kernel
Ktr<-koplsKernel(Xtr,NULL,'g',sigma)

## Center kernel
Ktr_centered<-koplsCenterKTrTr(Ktr)
```

`koplsConfusionMatrix` *Calculation of confusion matrix*

Description

Calculates a confusion matrix from classification results.

Usage

```
koplsConfusionMatrix(true, pred)
```

Arguments

`true`	True class belongings.
`pred`	Predicted class belongings.

Value

The confusion matrix.

Author(s)

Max Bylesjo and Mattias Rantalainen

References

Rantalainen M, Bylesjo M, Cloarec O, Nicholson JK, Holmes E and Trygg J. **Kernel-based orthogonal projections to latent structures (K-OPLS)**, *J Chemometrics* 2007; 21:376-385. doi:10.1002/cem.1071.

Examples

`koplsCrossValSet` *Generate training/test observations for cross-validation*

Description

Generates sets of training/test observations useful for cross-validation (CV). How the sets are generated is determined by the `type` parameter, which can be either 'nfold' for n-fold cross-validation, 'mccv' for Monte Carlo CV, 'mccvb' for Monte Carlo class-balanced CV.

Usage

```
koplsCrossValSet(K, Y, type = "nfold", nfold, i, trainFrac = (2/3))
```

Arguments

`K`	Kernel matrix.
`Y`	Response matrix.
`type`	Type of cross-validation: 'nfold' for n-fold, 'mccv' for Monte Carlo CV, 'mccvb' for Monte Carlo class-balanced CV.
`nfold`	Number of total nfold rounds (if type='nfold').
`i`	Current nfold round (if type='nfold').
`trainFrac`	Fraction of observations in training set.

Details

If `type` is set to 'nfold', the parameter `nfold` determines the number of rounds, which are later subindexed by the `i` parameter. If 'mccv' or 'mccvb', the `trainFrac` parameter determines the fraction of observations that will belong to the training set; remaining observations end up in the test set.

Value

List object with the following entries:

`KTrTr`	Kernel training matrix; KTrTr = <phi(Xtr),phi(Xtr)>.
`KTeTr`	Kernel test/training matrix; KTeTr = <phi(Xte), phi(Xtr)>.
`KTeTe`	Kernel test matrix; KTeTe = <phi(Xte),phi(Xte)>.
`yTrain`	Y training set.
`yTest`	Y test set.
`trainInd`	Indices of training set observations.
`testInd`	Indices of test set observations.

Author(s)

Max Bylesjo and Mattias Rantalainen

References

Rantalainen M, Bylesjo M, Cloarec O, Nicholson JK, Holmes E and Trygg J. **Kernel-based orthogonal projections to latent structures (K-OPLS)**, *J Chemometrics* 2007; 21:376-385. doi:10.1002/cem.1071.

Examples

`koplsDemo`	*K-OPLS demonstration procedure*

Description

This script contains a demonstration of the functionality in the `kopls` package using a simulated data set.

Usage

```
demo(koplsDemo)
```

Details

The data set is represented by 1000 spectral variables from two different classes and is available in the an attached data set. The demonstration essentially consists of two main steps.

The first step is to demonstrate how K-OPLS handles the model evaluation (using cross-validation), model building and subsequent classification of external data from a non-linear data set. The second step is to demonstrate how K-OPLS works in the presence of response-independent (`Y`-orthogonal) variation, using the same data set but with a strong systematic class-specific disturbance added.

The `koplsExample` data set contains the following objects:

`Xtr`	The training data matrix, with 400 observations and 1000 spectral variables.
`Xte`	The test data matrix, with 400 observations and 1000 spectral variables.
`Xtro`	Same data as 'Xtr', but with class-specific systematic noise added.
`Xteo`	Same data as 'Xte', but with class-specific systematic noise added.
`Ytr`	A binary matrix of class assignments for the training data.
`Yte`	A binary matrix of class assignments for the test data.
`pch.vec`	A vector with character indices (for plotting).
`col.vec`	A vector with colors (for plotting).

Author(s)

Max Bylesjo and Mattias Rantalainen

References

Rantalainen M, Bylesjo M, Cloarec O, Nicholson JK, Holmes E and Trygg J. **Kernel-based orthogonal projections to latent structures (K-OPLS)**, *J Chemometrics* 2007; 21:376-385. doi:10.1002/cem.1071.

`koplsDummy`	*Convertion of integer vector to dummy matrix*

Description

Converts integer vector to binary class matrix ('dummy' matrix).

Usage

```
koplsDummy(class)
```

Arguments

`class`	Integer vector containing values denoting class belonging.
`numClasses`	Number of classes. If NA, the number of unique entries in `class` will be used instead.

Value

A matrix with rows corresponding to observations and columns to classes. Each element in matrix is either one (observation belongs to class) or zero (observation does not belong to class).

Author(s)

Max Bylesjo and Mattias Rantalainen

References

Rantalainen M, Bylesjo M, Cloarec O, Nicholson JK, Holmes E and Trygg J. **Kernel-based orthogonal projections to latent structures (K-OPLS)**, *J Chemometrics* 2007; 21:376-385. doi:10.1002/cem.1071.

Examples

`koplsKernel`	*Kernel construction method*

Description

Constructs a kernel matrix K = <phi(`X1`), phi(`X2`)>. The kernel function k() determines how the data is transformed and is passed as the separate parameter `Ktype` to the function. Currently `Ktype` can be either 'g' (Gaussian) or 'p' (polynomial); see the supplied reference for definitions of these kernel functions.

Usage

```
koplsKernel(X1, X2, Ktype, param)
```

Arguments

`X1`	'Left side' matrix in expression K = <phi(`X1`), phi(`X2`)>.
`X2`	'Right side' matrix in expression K = <phi(`X1`), phi(`X2`)>.
`Ktype`	Type of kernel function: either 'g' (Gaussian) or 'p' (polynomial).
`param`	A vector with parameters to the kernel function.

Details

If the second parameter `X2` is set to NULL, the kernel matrix is considered to be symmetric and hence the kernel function can be applied at a considerable speed reduction. , This applies generally to pure training kernel or test kernels (where `X1` = `X2`),

but not to a hybrid test/training kernel (where `X1` != `X2`).

Value

The kernel matrix K, transformed by the kernel function specified by `Ktype`.

Author(s)

Max Bylesjo and Mattias Rantalainen

References

Rantalainen M, Bylesjo M, Cloarec O, Nicholson JK, Holmes E and Trygg J. **Kernel-based orthogonal projections to latent structures (K-OPLS)**, *J Chemometrics* 2007; 21:376-385. doi:10.1002/cem.1071.

Examples

```
data(koplsExample)

## Define kernel function parameter
sigma<-25

## Construct kernels
Ktr<-koplsKernel(Xtr,NULL,'g',sigma)
KteTr<-koplsKernel(Xte,Xtr,'g',sigma)
KteTe<-koplsKernel(Xte,NULL,'g',sigma)
```

`koplsMaxClassify` *Classification rule based on the maximum class belonging*

Description

Classification function that determines class belonging based on the maximum value of Yhat for each class.

Usage

```
koplsMaxClassify(data)
```

Arguments

`data`	Matrix of predicted class values (Yhat) for each class.

Value

A vector of class belongings (integer values).

Author(s)

Max Bylesjo and Mattias Rantalainen

References

Rantalainen M, Bylesjo M, Cloarec O, Nicholson JK, Holmes E and Trygg J. **Kernel-based orthogonal projections to latent structures (K-OPLS)**, *J Chemometrics* 2007; 21:376-385. doi:10.1002/cem.1071.

Examples

`koplsModel` *K-OPLS model training*

Description

Function for training a K-OPLS model. The function constructs a predictive regression model for predicting the values of `Y` by using the information in `K`. The explained variation is separated into predictive components, which dimensionality is determined by the parameter `A`, and `Y`-orthogonal components; dimensionality determined by the parameter `nox`.

Usage

```
koplsModel(K, Y, A, nox, preProcK = "mc", preProcY = "mc")
```

Arguments

`K`	Kernel matrix (un-centered); K = <phi(Xtr),phi(Xtr)>
`Y`	Response matrix (un-centered/scaled).
`A`	Number of predictive components.
`nox`	Number of `Y`-orthogonal components. ,
`preProcK`	Pre-processing parameters for the `K` matrix: 'mc' for mean-centering, 'no' for no centering. ,
`preProcY`	Pre-processing parameters for the `Y` matrix: 'mc' for mean-centering, 'uv' for mc + scaling to unit variance, 'pa' for mc + Pareto, 'no' for no scaling.

Details

Value

List with the following components:

`Cp`	`Y` loading matrix.
`Sp`	Sigma matrix, containing singular values from `Y'*K*Y` used for scaling.
`Sps`	Sp^(-1/2).
`Up`	`Y` score matrix.
`Tp`	Predictive score matrix for all `Y`-orthogonal components.
`T`	Predictive score matrix for the final `Y`-orthogonal component model.
`co`	`Y`-orthogonal loading vectors.
`so`	Eigenvalues from estimation of `Y`-orthogonal loading vectors.
`To`	`Y`-orthogonal score matrix.
`toNorm`	Norm of the `Y`-orthogonal score matrix prior to scaling.
`Bt`	T-U regression coefficients for predictions.
`A`	Number of predictive components.
`nox`	Number of `Y`-orthogonal components.
`K`	The kernel matrix.
`EEprime`	The deflated kernel matrix for residual statistics.
`sstot_K`	Total sums of squares in `K`
`R2X`	Cumulative explained variation for all model components.
`R2XO`	Cumulative explained variation for `Y`-orthogonal model components.
`R2XC`	Explained variation for predictive model components after addition of `Y`-orthogonal model components.
`sstot_Y`	Total sums of squares in `Y`.
`R2Y`	Explained variation of `Y`.
`preProc`	Pre-processing parameters:
K	Pre-processing setting for `K` = `preProcK`.
Y	Pre-processing setting for `Y` = `preProcY`.
paramsY	Scaling parameters for `Y`.

Author(s)

Max Bylesjo and Mattias Rantalainen

References

Rantalainen M, Bylesjo M, Cloarec O, Nicholson JK, Holmes E and Trygg J. **Kernel-based orthogonal projections to latent structures (K-OPLS)**, *J Chemometrics* 2007; 21:376-385. doi:10.1002/cem.1071.

Examples

```
## Load data set
data(koplsExample)

## Define kernel function parameter
sigma<-25

## Define number of Y-orthogonal components
nox<-3

## Construct kernel
Ktr<-koplsKernel(Xtr,NULL,'g',sigma)

## Model
model<-koplsModel(Ktr,Ytr,1,nox,'mc','mc');

## Visualize results
koplsPlotModelDiagnostics(model)
title("Model diagnostics without cross-validation")
```

`koplsPlotCVDiagnostics` *Overview plot of cross-validation results*

Description

Produces overview plots of cross-validation results retured from the `koplsCV` function.

Usage

```
koplsPlotCVDiagnostics(model.full, plot.values = FALSE)
```

Arguments

`model.full`	The cross-validation model result (see `koplsCV`).
`plot.values`	If TRUE, the exact values will be displayed on the bars as text labels.

Details

Produces 2x2 panels of bar plots, containing the total explained variation (R2X), the Y-orthogonal explained variation (R2XO), the Y-correlated explained variation (R2XC) and the predicted variation from cross-validation (Q2Y).

Author(s)

Max Bylesjo and Mattias Rantalainen

References

Rantalainen M, Bylesjo M, Cloarec O, Nicholson JK, Holmes E and Trygg J. **Kernel-based orthogonal projections to latent structures (K-OPLS)**, *J Chemometrics* 2007; 21:376-385. doi:10.1002/cem.1071.

Examples

```
## Load data set
data(koplsExample)

## Define kernel function parameter
sigma<-25

## Construct kernel
Ktr<-koplsKernel(Xtr,NULL,'g',sigma)

## Find optimal number of Y-orthogonal components by cross-validation
## The cross-validation tests models with Y-orthogonal components 0
through numYo
modelCV<-koplsCV(Ktr,Ytr,1,3,nrcv=7,cvType='nfold',preProcK='mc',
preProcY='mc',modelType='da')

## Visualize results
koplsPlotCVDiagnostics(modelCV)
title("Statistics from K-OPLS cross-validation of original data")
```

`koplsPlotModelDiagnostics` *Overview of model training results*

Description

Produces overview plots of model training results returned from the `koplsModel` function.

Usage

```
koplsPlotModelDiagnostics(model, plot.values = FALSE)
```

Arguments

`model`	The model training result (see `koplsModel`).
`plot.values`	If TRUE, the exact values will be displayed on the bars as text labels.

Details

Produces 2x2 panels of bar plots, containing the total explained variation (R2X), the Y-orthogonal explained variation (R2XO), the Y-correlated explained variation (R2XC) and (if available) the predicted variation from cross-validation (Q2Y).

Author(s)

Max Bylesjo and Mattias Rantalainen

References

Rantalainen M, Bylesjo M, Cloarec O, Nicholson JK, Holmes E and Trygg J. **Kernel-based orthogonal projections to latent structures (K-OPLS)**, *J Chemometrics* 2007; 21:376-385. doi:10.1002/cem.1071.

Examples

```
## Load data set
data(koplsExample)

## Define kernel function parameter
sigma<-25

## Define number of Y-orthogonal components
nox<-3

## Construct kernel
Ktr<-koplsKernel(Xtr,NULL,'g',sigma)

## Model
model<-koplsModel(Ktr,Ytr,1,nox,'mc','mc');

## Visualize results
koplsPlotModelDiagnostics(model)
title("Model diagnostics without cross-validation")
```

`koplsPlotScores` *Plots scores from trained K-OPLS models*

Description

, Produces score plots from K-OPLS models. If model components are unspecified,

all possible combinations are displayed as a scatter plot matrix. Otherwise, two selected components will be shown using a traditional 2D scatter plot.

Usage

```
koplsPlotScores(model, x = NA, xsub = "p", y = NA, ysub = "o")
```

Arguments

`model`	K-OPLS model (see `koplsModel`).
`x`	x-axis score vector index.
`xsub`	Identifying value for `x`: Either 'p' for predictive component or 'o' for Y-orthogonal component.
`y`	y-axis score vector index.
`ysub`	Identifying value for `y`: Either 'p' for predictive component or 'o' for Y-orthogonal component .

Details

The diagnol of the scatter plot matrix depicts the kernel density of that particular score vector. Any additional parameters will be passed on to the plot() function, which can be used to e.g. set the color or shape of the displayed data points.

Author(s)

Max Bylesjo and Mattias Rantalainen

References

Rantalainen M, Bylesjo M, Cloarec O, Nicholson JK, Holmes E and Trygg J. **Kernel-based orthogonal projections to latent structures (K-OPLS)**, *J Chemometrics* 2007; 21:376-385. doi:10.1002/cem.1071.

Examples

```
## Load data set
data(koplsExample)

## Define kernel function parameter
sigma<-25

## Define number of Y-orthogonal components
nox<-3

## Construct kernel
Ktr<-koplsKernel(Xtr,NULL,'g',sigma)

## Model
model<-koplsModel(Ktr,Ytr,1,nox,'mc','mc');

#### Visualize results

## Shows all scores as scatter plot matrix
## col.vec defines class colors and is loaded by data(koplsExample)
koplsPlotScores(model, col=col.vec)

## Shows tp1 vs to1, colored by class
## pch.vec defines class glyph types and is loaded by data(koplsExample)
koplsPlotScores(model, x=1, xsub='p', y=1, ysub='o', col=col.vec,
 pch=pch.vec)

## Shows to1 vs to2
koplsPlotScores(model, x=1, xsub='o', y=2, ysub='o', col=col.vec,
 pch=pch.vec)
```

`koplsPlotSensSpec` *Plots sensitivity and specificity results from cross-validation*

Description

Plots sensitivity and specificity results from cross-validation in a bar plot. The produced bars are shown separately for each class including overall sensitivity and specificity results.

Usage

```
koplsPlotSensSpec(modelFull)
```

Arguments

`modelFull` The 'koplscv' model from cross-validation (see `koplsCV`).

Value

The resulting sensitivity and specificity measures.

Author(s)

Max Bylesjo and Mattias Rantalainen

References

Rantalainen M, Bylesjo M, Cloarec O, Nicholson JK, Holmes E and Trygg J. **Kernel-based orthogonal projections to latent structures (K-OPLS)**, *J Chemometrics* 2007; 21:376-385. doi:10.1002/cem.1071.

Examples

```
## Load data set
data(koplsExample)

## Define kernel function parameter
sigma<-25

## Construct kernel
Ktr<-koplsKernel(Xtr,NULL,'g',sigma)

## Find optimal number of Y-orthogonal components by cross-validation
## The cross-validation tests models with Y-orthogonal components 0
through numYo
modelCV<-koplsCV(Ktr,Ytr,1,3,nrcv=7,cvType='nfold',preProcK='mc',
preProcY='mc',modelType='da')

## Visualize results
koplsPlotSensSpec(modelCV)
```

`koplsPredict` *Prediction of new samples from a K-OPLS model*

Description

Performs prediction of new samples from an existing K-OPLS model (see `koplsModel`). The function projects the Y-predictive and Y-orthogonal scores components to predict a value of the response matrix Y. The dimensionality of the parameters is determined from `model`.

Usage

```
koplsPredict(KteTr, Ktest, Ktrain, model, nox = NA, rescaleY = FALSE)
```

Arguments

	,
	The hybrid test/training kernel matrix; KteTr = <phi(Xte), phi(Xtr)>.
`KteTe` `KteTr`	The test kernel matrix; Ktest = <phi(Xte),phi(Xte)>. ,
`Ktrain`	The training kernel matrix (same as used in model training); Ktrain = <phi(Xtr), phi(Xtr)>
`model`	The trained K-OPLS model (see `koplsModel`).
`nox`	Number of Y-orthogonal score vectors. If undefined, the value used in `model` will be employed.
`rescaleY`	If true, the estimated Yhat values will be rescaled according to the scaling parameters in `model`. Otherwise Yhat values will be returned as is (default).

Value

`Tp`	Predicted predictive score matrix for all generations 0:`nox` of Y-orthogonal vectors.
`T`	Predictive score matrix for the final model with `nox` Y-orthogonal vectors.
`to`	Predicted Y-orthogonal score vectors. ,
`EEprime`	Calculated residuals for the test kernel matrix `Ktest`, useful e.g. for residual statistics.
`Yhat`	Predicted values of the response matrix.

Author(s)

Max Bylesjo and Mattias Rantalainen

References

Rantalainen M, Bylesjo M, Cloarec O, Nicholson JK, Holmes E and Trygg J. **Kernel-based orthogonal projections to latent structures (K-OPLS)**, *J Chemometrics* 2007; 21:376-385. doi:10.1002/cem.1071.

Examples

```
## Load data set
data(koplsExample)

## Define kernel function parameter
sigma<-25

## Define number of Y-orthogonal components
nox<-3

## Construct kernels
Ktr<-koplsKernel(Xtr,NULL,'g',sigma)
KteTr<-koplsKernel(Xte,Xtr,'g',sigma)
KteTe<-koplsKernel(Xte,NULL,'g',sigma)

## Model
```

```
model<-koplsModel(Ktr,Ytr,1,nox,'mc','mc');

## Predict
modelPred<-koplsPredict(KteTr,KteTe,Ktr,model,rescaleY=TRUE)

## Visualize
plot(modelPred$Yhat, Yte, xlab="Predicted", ylab="Observed")
abline(v=0.5, col="Red", lty=2) ## Approximate decision boundary
```

`koplsReDummy` *Reconstruct class vector*

Description

Reconstruncts a (integer) class vector from a binary (dummy) matrix.

Usage

```
koplsReDummy(dummy)
```

Arguments

`dummy`	Dummy matrix. See `koplsDummy` for details.

Value

The reconstructed integer class vector.

Author(s)

Max Bylesjo and Mattias Rantalainen

References

Rantalainen M, Bylesjo M, Cloarec O, Nicholson JK, Holmes E and Trygg J. **Kernel-based orthogonal projections to latent structures (K-OPLS)**, *J Chemometrics* 2007; 21:376-385. doi:10.1002/cem.1071.

Examples

`koplsRescale`	*Matrix scaling based on pre-defined parameters*

Description

Scales a matrix based on pre-defined parameters from a scaling object.

Usage

```
koplsRescale(model, x = NA)
```

Arguments

`model`	Scaled object list, see `link{koplsScale}` for details.
`x`	The matrix to be scaled. If NA, the object contained in `model` will be scaled and returned.

Value

The rescaled matrix.

Author(s)

Max Bylesjo and Mattias Rantalainen

References

Rantalainen M, Bylesjo M, Cloarec O, Nicholson JK, Holmes E and Trygg J. **Kernel-based orthogonal projections to latent structures (K-OPLS)**, *J Chemometrics* 2007; 21:376-385. doi:10.1002/cem.1071.

Examples

`koplsScale`	*Matrix scaling function*

Description

Function for mean-centering and scaling of a matrix.

Usage

```
koplsScale(x, center = "mc", scale = "none")
```

Arguments

`x`	The matrix to be mean-centered and/or scaled.
`center`	Mean-centering type: Either 'mc' for column-wise mean-centering or 'no' for no mean-centering.
`scale`	Scaling type: 'uv' for scaling to unit variance, 'pareto' for Pareto scaling (sqrt(uv)) or 'no' for no scaling.

Value

A list with the following properties:

`x`	The scaled matrix.
`meanVector`	Vector with mean values (possibly) used in the scaling.
`sdVector`	Vector with standard deviation values (possibly) used in the scaling.
`scale`	Scaling type: see `link{koplsScale}` for details.
`center`	Mean-centering type see `link{koplsScale}` for details.

Author(s)

Max Bylesjo and Mattias Rantalainen

References

Rantalainen M, Bylesjo M, Cloarec O, Nicholson JK, Holmes E and Trygg J. **Kernel-based orthogonal projections to latent structures (K-OPLS)**, *J Chemometrics* 2007; 21:376-385. doi:10.1002/cem.1071.

Examples

`koplsScaleApply` *Apply matrix scaling*

Description

Applies matrix scaling from an external scaling object and returns the scaled matrix with scaling parameters.

Usage

```
koplsScaleApply(model, x)
```

Arguments

`model`	Scaled object list, see `link{koplsScale}` for details.
`x`	The matrix to be scaled. If NA, the object contained in `model` will be scaled and returned.

Value

A list with the following properties:

`x`	The scaled matrix.
`meanVector`	Vector with mean values (possibly) used in the scaling.
`sdVector`	Vector with standard deviation values (possibly) used in the scaling.
`scale`	Scaling type: see `link{koplsScale}` for details.
`center`	Mean-centering type see `link{koplsScale}` for details.

Author(s)

Max Bylesjo and Mattias Rantalainen

References

Rantalainen M, Bylesjo M, Cloarec O, Nicholson JK, Holmes E and Trygg J. **Kernel-based orthogonal projections to latent structures (K-OPLS)**, *J Chemometrics* 2007; 21:376-385. doi:10.1002/cem.1071.

Examples

`koplsSensSpec` *Sensitivity and specificity calculations for classification*

Description

Calculates sensitivity and specificity values for classification in a class-wise fashion.

Usage

```
koplsSensSpec(trueClass, predClass)
```

Arguments

`trueClass`	Matrix of true class identifiers (integer).
`predClass`	Matrix of predicted class identifiers (integer).

Value

A list with the following properties:

`TPtot`	Total true positive (TP) rate.
`FPtot`	Total false positive (FP) rate.
`TNtot`	Total true negative (TN) rate.
`FNtot`	Total false negative (FN) rate.
`sensTot`	Overall sensitivity.
`specTot`	Overall specificity.

`TP`	TP rate for each class.
`FP`	FP rate for each class.
`TN`	TN rate for each class.
`FN`	FN rate for each class.
`sens`	Sensitivity for each class.
`spec`	Specificity for each class.
`Ntot`	Total number of entries.

Author(s)

Max Bylesjo and Mattias Rantalainen

References

Rantalainen M, Bylesjo M, Cloarec O, Nicholson JK, Holmes E and Trygg J. **Kernel-based orthogonal projections to latent structures (K-OPLS)**, *J Chemometrics* 2007; 21:376-385. doi:10.1002/cem.1071.

Examples

References

[1] Rantalainen M, Bylesjo M, Cloarec O, Nicholson JK, Holmes E and Trygg J. **Kernel-based orthogonal projections to latent structures (K-OPLS)**. *J Chemometrics* 2007; 21:376-385.
