# Supplementary material for: K-OPLS package: Kernel-based orthogonal projections to latent structures for prediction and interpretation in feature space
Source: BMC Bioinformatics. 2008 Feb 19;9:106. doi: 10.1186/1471-2105-9-106 (PMC2323673; doi:10.1186/1471-2105-9-106)
Supplement: Additional File 4 — K-OPLS package version 1.0.3 for MATLAB. Provides the K-OPLS version 1.0.3 source code and documentation for MATLAB [file 1471-2105-9-106-S4.zip › doc/koplsVignette.pdf]

Kernel-based Orthogonal Projections to Latent Structures (K-OPLS)

Max Bylesjö and Mattias Rantalainen

January 23, 2008

Abstract

This document describes features of the `kopls` package for MATLAB. The presented package provides an open-source, platform-independent implementation of the Kernel-based Orthogonal Projections to Latent Structures (K-OPLS) method; a kernel-based classification and regression method. In relation to other kernel-based methods, K-OPLS offers unique properties facilitating separate modeling of predictive variation and structured noise in the feature space. While providing prediction results similar to other kernel-based methods, K-OPLS features enhanced interpretational capabilities; allowing detection of unanticipated systematic variation in the data such as instrumental drift, batch variability or unexpected biological variation.

Contents

1 Features 3

2 Requirements 3

3 Installation 3

4 Getting started 4

5 Availability 4

6 Specification of functions 4

6.1 koplsBasicClassify 4
6.2 koplsCV 5
6.3 koplsCenterKTeTe 6
6.4 koplsCenterKTeTr 7
6.5 koplsCenterKTrTr 7
6.6 koplsConfusionMatrix 8
6.7 koplsCrossValSet 8
6.8 koplsDummy 9
6.9 koplsKernel 9
6.10 koplsMaxClassify 10
6.11 koplsModel 11
6.12 koplsPlotCVDiagnostics 12
6.13 koplsPlotModelDiagnostics 12
6.14 koplsPlotScores 13
6.15 koplsPlotSensSpec 13
6.16 koplsPredict 14
6.17 koplsReDummy 15
6.18 koplsRescale 15
6.19 koplsScale 16
6.20 koplsScaleApply 16
6.21 koplsSensSpec 17
6.22 koplsDemo 18

1 Features

The package features the following main functionality:

1. Estimation (training) of K-OPLS models.
2. Prediction of new data using the estimated K-OPLS model in the preceding step.
3. Cross-validation functionality to estimate the generalization error of a K-OPLS model. This is intended to guide the selection of the number of Y-predictive components A and the number of Y-orthogonal components A_o. The supported implementations are:
   - n-fold cross-validation.
   - Monte Carlo Cross-Validation (MCCV)
   - Monte Carlo Class-balanced Cross-Validation (for discriminant analysis cases).
4. Kernel functions, including the polynomial and Gaussian kernel functions.
5. Model statistics:
   - The explained variation of X (R^2_X).
   - The explained variation of Y (R^2_Y).
   - Prediction statistics over cross-validation for regression tasks (Q^2_Y, which is inversely proportional to the generalization error).
   - Prediction statistics over cross-validation for classification tasks (sensitivity and specificity measures).
6. Plot functions for visualization:
   - Scatter plot matrices for model score components.
   - Model statistics and diagnostics plots.

2 Requirements

A functional installation of MATLAB 7.0 or later is required.

To get full functionality for the `koplsPlotScores()` function, the MATLAB statistics toolbox is required (see documentation on this function for further details).

3 Installation

Unzip the MATLAB source files (`*.m`) into any directory of choice. Inside MATLAB, set the path to include the extracted files.

4 Getting started

The package includes a demonstration of the functionality available in the package, which is intended to be a starting point for future use. The demonstration is based on a simulated data set, represented by 1000 spectral variables from two different classes and is available in a supplied workspace. The demonstration essentially consists two main steps.

The first step is to demonstrate how K-OPLS handles the model evaluation (using cross-validation), model building and subsequent classification of external data from a non-linear data set.

The second step is to demonstrate how K-OPLS works in the presence of response-independent (Y-orthogonal) variation, using the same data set but with a strong systematic class-specific disturbance added.

To run the demonstration:

1. Make sure that all included `kopls*.m` files are in the current path
2. Run the demo by typing `koplsDemo`.

5 Availability

The K-OPLS package for MATLAB is freely available for download at `http://kopls.sourceforge.net/`.

6 Specification of functions

6.1 koplsBasicClassify

```
function [predClass]=koplsBasicClassify(data,k)


Classification function that assesses class belonging of a predicted
response in 'data' based on a fixed threshold 'k'.

** INPUT
data = matrix containing the predicted response matrix Y,
  where columns denote classes and rows observations.
k = threshold value used to assign class categories.

** OUTPUT
predClass = the predicted class(es) of 'data' given 'k'.
```

6.2 koplsCV

```
function [modelMain]=koplsCV(K,Y,A,oax,nrcv,cvType,preProcK,preProcY,
cvFrac,modelType,verbose)


Function for performing K-OPLS cross-validation for a set of
Y-orthogonal components. The function returns a number of diagnostic
parameters which can be used to determine the optimal number
of model components.

** INPUT
K = The kernel matrix (un-centered); see 'koplsKernel()' for details.
Y = The response matrix (un-centered/scaled). Could be binary (for
  discriminant analysis) or real-valued.
A = The number of Y-predictive components (integer).
oax = The number of Y-orthogonal components (integer).
nrcv = Number of cross-validation rounds (integer).
cvType = Type of cross-validation. Either 'nfold' for n-fold
  cross-validation, 'mccv' for Monte Carlo CV or 'mccvb' for
  Monte Carlo class-balanced CV. See also 'koplsCrossValSet()' for
  details.
preProcK = Pre-processing settings for the kernel matrix.
  Either 'mc' for mean-centering or 'no' for no pre-processing.
preProcY = Pre-processing parameter for Y. Either 'mc' for
  mean-centering, 'uv' for mc + scaling to unit-variance,
  'pareto' for mc + Pareto-scaling or 'no' for no scaling.
cvFrac = Fraction of observations in the training set during
  cross-validation. Only applicable for 'mccv' or 'mccvb'
  cross-validation (see 'cvType').
modelType = 'da' for discriminant analysis, 're' for regression.
  If 'da', sensitivity and specificity will be calculated.
verbose = If zero, no output will be displayed, otherwise some
  output will be displayed regarding the cross-validation progress
   (default).

** OUTPUT
modelMain = Object with 'A' predictive components and 'oax'
  Y-orthogonal components. Contains the following entries:
 cv = Cross-validation results:
     Q2Yhat = Total Q-square result for all Y-orthogonal components.
 Q2YhatVars = Q-square result per Y-variable for all Y-orthogonal
      components.
 Yhat = All predicted Y values as a concatenated matrix.
```

```
 Tcv = Predictive score vector T for all cross-validation rounds.
 cvTrainIndex = Indices for the training set observations during
      the cross-validation rounds.
 cvTestIndex = Indices for the test set observations during the
      cross-validation rounds.
 da = Cross-validation results specifically for discriminant
      analysis (DA) cases:
   predClass = Predicted class list per class and Y-orthogonal
      components (integer values).
 trueClass = Predicted class list per class and Y-orthogonal
      components (integer values).
 sensSpec = Sensitivity and specificity values per class and
      Y-orthogonal components (integer values).
 confusionMatrix = Confusion matrix during cross-validation
      rounds.
 nclasses = Number of classes in model.
 decisionRule = Decision rule used: 'max' or 'fixed'.
 args = Arguments to the function:
     A = Number of Y-predictive components.
 oax = Number of Y-orthogonal components.
```

6.3 koplsCenterKTeTe

```
function [KteTe]=koplsCenterKTeTe(KteTe,KteTr,KtrTr)
```

```
Centering function for the test kernel, which is constructed
from the test matrix Xte as KteTe = <phi(Xte), phi(Xte)>.
Requires additional (un-centered) kernels KteTr and KteTr to
estimate mean values (see 'koplsKernel()' for details on
constructing a kernel matrix).

** INPUT
KteTe = Test kernel matrix; KteTe = <phi(Xte), phi(Xte)>.
KteTr = Test/training kernel matrix;
  KteTr = <phi(Xte), phi(Xtr)>.
KtrTr = Training kernel matrix; KtrTr = <phi(Xtr), phi(Xtr)>.

** OUTPUT
KteTe = The centered test kernel matrix.
```

6.4 koplsCenterKTeTr

```
function [KteTr]=koplsCenterKTeTr(KteTr,KtrTr)
```

```
Centering function for the hybrid test/training kernel, which
is constructed from the test matrix Xte and the training matrix
Xtr as KteTr = <phi(Xte), phi(Xtr)>. Requires additional
(un-centered) training kernel to estimate mean values
(see 'koplsKernel()' for details on constructing a kernel matrix).

** INPUT
KteTr = Hybrid test/training kernel matrix;
  KteTr = <phi(Xte), phi(Xtr)>.
KtrTr = Training kernel matrix; Ktrain = <phi(Xtr), phi(Xtr)>.

** OUTPUT
KteTr = The centered kernel matrix.
```

6.5 koplsCenterKTrTr

```
function [K]=koplsCenterKTrTr(K)
```

```
Centering function for the training kernel, which is constructed
from the training matrix Xtr as K = <phi(Xtr), phi(Xtr)>
(see 'koplsKernel()' for details on constructing a kernel matrix).

** INPUT
K = Training kernel matrix; K = <phi(Xtr), phi(Xtr)>.

** OUTPUT
K = The centered kernel matrix.
```

6.6 koplsConfusionMatrix

```
function [A]=koplsConfusionMatrix(true,pred)
```

```
Calculates a confusion matrix from classification results.

** INPUT
true = true class belonging.
pred = predicted class assignment.

** OUTPUT
A = Confusion matrix.
```

6.7 koplsCrossValSet

```
function [cvSet]=koplsCrossValSet(K,Y,modelFrac,type,nfold,nfoldRound)
```

```
Generates sets of training/test observations useful for cross-
validation (CV). How the sets are generated is determined by the
'type' parameter, which can be either 'nfold' for n-fold cross-
validation, 'mccv' for Monte Carlo CV, 'mccvb' for Monte Carlo
class-balanced CV.

** INPUT
K = Kernel matrix.
Y = Response matrix.
type = Type of cross-validation:
'nfold' for n-fold, 'mccv' for Monte Carlo CV, 'mccvb' for
Monte Carlo class-balanced CV.
 nfold = Number of total nfold rounds (if type='nfold').
 nfoldRound = Current nfold round (if type='nfold').

** OUTPUT
 Object 'cvSet' with the following entries:
  type = Cross-validation type.
  nfold = Number of nfold rounds.
  nfoldRound = The current nfold round.
  KTrTr = Kernel training matrix; KTrTr = <phi(Xtr),phi(Xtr)>.
```

```
  KTeTr = Kernel test/training matrix;
      KTeTr = <phi(Xte),phi(Xtr)>.}
  KTeTe = Kernel test matrix; KTeTe = <phi(Xte),phi(Xte)>.
  yTraining = Y training set.
  yTest = Y test set.
  trainingIndex = Indices of training set observations.
  testIndex = Indices of test set observations.
```

6.8 koplsDummy

```
function [dummy, labels_sorted]=koplsDummy(class, numClasses);


Converts integer vector to binary matrix (dummy matrix).

** INPUT
class = vector with class belongings (integer).
numClasses = pre-defines the number of classes in the output
(if undefined, the number of unique entries in 'class' will be
  used).

** OUTPUT
dummy = A matrix with rows corresponding to observations and
  columns to classes. Each element in matrix is either one
  (observation belongs to class) or zero (observation does not
  belong to class).
labels_sorted = The class labels that are found in class in
sorted order.
```

6.9 koplsKernel

```
function [K]=koplsKernel(X1,X2,Ktype,params)


Constructs a kernel matrix K = <phi(X1), phi(X2)>.
The kernel function k() determines how the data is transformed and
```

```
is passed as the separate parameter 'Ktype' to the function.
Currently 'Ktype' can be either 'g' (Gaussian) or 'p' (polynomial).

** INPUT
X1 = the first X matrix (non-centered). This is the left side in
  the expression K = <phi(X1), phi(X2)>.
X2 = the second X matrix (non-centered). This is the right side
  in the expression K = <phi(X1), phi(X2)>.
 If X2 = [] (empty set), then only X1 will be used for
 the calculations. This way, only (n^2 - n)/2 instead of n^2
 calculations have to be performed, which is typically much
 faster. Only applicable for pure training or testing kernels.
Ktype = the type of kernel used. Supported entries are:
  - 'g': Gaussian kernel.
  - 'p': Polynomial kernel.
params = A vector with parameter for the kernel function.
  (Currently, all supported kernel functions use a scalar value
   so the vector property of the parameters is for future
   compability).

** OUTPUT
K = The kernel matrix, transformed by the kernel function
  specified by 'Ktype'.
```

6.10 koplsMaxClassify

```
function [predClass]=koplsMaxClassify(data)


Classification function that assesses class belonging of 'data'
based on the maximum value.

** INPUT
data = matrix containing the predicted response matrix Y,
  where columns denote classes and rows observations.

** OUTPUT
predClass = the predicted class(es) of 'data'.
```

6.11 koplsModel

```
function [model]=koplsModel(K,Y,A,nox,preProcK,preProcY)


Function for training a K-OPLS model. The function constructs a
predictive regression model for predicting the values of ’Y by
using the information in ’K’. The explained variation is separated
into predictive components (dimensionality is determined by the
parameter ’A’) and ’Y’-orthogonal components (dimensionality
determined by the parameter ’nox’).

** INPUT
K = Kernel matrix (un-centered); K = <phi(Xtr),phi(Xtr)>.
Y = Response matrix (un-centered/scaled).
A = Number of predictive components.
nox = Number of Y-orthogonal components.
preProcK = Pre-processing parameters for the ’K’ matrix:
  ’mc’ for mean-centering, ’no’ for no centering.
preProcY = Pre-processing parameters for the ’Y’ matrix:
  ’mc’ for mean-centering, ’uv’ for mc + scaling to unit variance,
  ’pa’ for mc + Pareto, ’no’ for no scaling.

** OUTPUT
model = Object with the following entries:
  Cp = Y loading matrix.
  Sp = Sigma matrix, containing singular values from Y’*K*Y used
     for scaling.
  Sps = Sp^(-1/2).
  Up = Y score matrix.
  Tp = Predictive score matrix for all Y-orthogonal components.
  T = Predictive score matrix for the final model.
  co = Y-orthogonal loading vectors.
  so = Eigenvalues from estimation of Y-orthogonal loading vectors.
  To = Y-orthogonal score matrix.
  toNorm = Norm of the Y-orthogonal score matrix prior to scaling.
  Bt = T-U regression coefficients for predictions.
  A = Number of predictive components.
  nox = Number of Y-orthogonal components.
  K = The kernel matrix.
  EEprime = The deflated kernel matrix for residual statistics.
  sstot_K = Total sums of squares in ’K’.
  R2X = Cumulative explained variation for all model components.
  R2XO = Cumulative explained variation for Y-orthogonal
     model components.
  R2XC = Explained variation for predictive model components after
     addition of Y-orthogonal model components.
  sstot_Y = Total sums of squares in Y.
```

```
  R2Y = Explained variation of Y.
  preProc = Pre-processing parameters:
      K = Pre-processing setting for K = 'preProcK'.
  Y = Pre-processing setting for Y = 'preProcY'.
  paramsY = Scaling parameters for Y.
```

6.12 koplsPlotCVDiagnostics

```
function []=koplsPlotCVDiagnostics(modelFull)


Plots diagnostic parameters from K-OPLS cross-validation.
This includes:
- R2X = Cumulative explained variation for all model components.
- R2Xortho = Cumulative explained variation for all Y-orthogonal
   model components.
- R2Xcorr = Explained variation for predictive model components
   after addition of Y-orthogonal model components.
- Q2Y = Total Q-square result (1 - pred. residual / original var.)
  for all Y-orthogonal components.

For further information regarding the definitation and calculation
of these quantities, see e.g the appendix of:
 * Trygg J and Wold S. J Chemometrics 2003; 17:53-64.

** INPUT
model = a model constructed using 'koplsModel()'.
```

6.13 koplsPlotModelDiagnostics

```
function []=koplsPlotModelDiagnostics(model)


Plots diagnostic parameters from K-OPLS cross-validation.
This includes:
- R2X = Cumulative explained variation for all model components.
```

```
- R2XO = Cumulative explained variation for all Y-orthogonal model
  components.
- R2XC = Explained variation for predictive model components after
      addition of Y-orthogonal model components.

For further information regarding the definitation and calculation
of these quantities, see e.g appendix of:
 * Trygg J and Wold S. J Chemometrics 2003; 17:53-64.

** INPUT
model = a model constructed using 'koplsModel()'.
```

6.14 koplsPlotScores

```
function []=koplsPlotScores(model,x,xsub,y,ysub)


Produces score plots from K-OPLS models. If model components are
unspecified, all possible combinations are displayed as a scatter
plot matrix (default). Otherwise, two selected components will be
shown using a traditional 2D scatter plot.

NB: The diagonal of the scatter plot matrix requires functionality
from the 'MATLAB statistics toolbox' to produce variable densities.
In the absence of the toolbox, the diagonal will consist of blank
plots.

** INPUT
model = the K-OPLS model generated using 'koplsModel()'.
x = the vector index for the x axis.
xsub = the vector identifier {'p', 'o'} for the x axis.
y = the vector index for the y axis.
ysub = the vector identifier {'p', 'o'} for the y axis
```

6.15 koplsPlotSensSpec

```
function [res]=koplsPlotSensSpec(modelFull)

Plots sensitivity and specificity results from cross-validation
in a bar plot. The produced bars are shown separately for each
class including overall sensitivity and specificity results.

** INPUT
modelFull = the K-OPLS cross-validation results from 'koplsCV()'

** OUTPUT
res = The resulting sensitivity and specificity measures.
```

6.16 koplsPredict

```
function [modelp]=koplsPredict(KteTr,Ktest,Ktrain,model,nox,rescaleY)


Performs prediction of new samples from an existing K-OPLS model
(see koplsModel()' to calculate K-OPLS models).
The function projects the Y-predictive and Y-orthogonal scores
components to predict a value of the response matrix Y.
The dimensionality of the parameters is determined from the
specified model.

** INPUT
KteTr = The hybrid test/training kernel matrix;
  KteTr = <phi(Xte),phi(Xtr)>.
Ktest = The pure test kernel matrix;
  Ktest = <phi(Xte),phi(Xte)>.
Ktrain = The training kernel matrix (same as used in
  model training); Ktrain = <phi(Xtr),phi(Xtr)>.
model = K-OPLS model object.
nox = Number of Y-orthogonal components. If not specified, the
  number used during model training will be employed.
rescaleY = Boolean parameter. If true, predicted values of the
  response (Yhat) is rescaled according to the pre-processing
  settings of the model. If false, Yhat is not rescaled (default).

** OUTPUT:
modelp = Object with the following entries:
  Tp = Predicted predictive score matrix for all generations
      0:'nox' of Y-orthogonal vectors.
  T = Predictive score matrix for the final model with 'nox'
```

```
    Y-orthogonal vectors.
to = Predicted Y-orthogonal score vectors.
EEprime = Calculated residuals for the test kernel 'Ktest',
    useful e.g. for residual statistics.
Yhat = Predicted values of the response matrix.
```

6.17 koplsReDummy

```
function [classVect]=koplsReDummy(Y)


Reconstructs a (integer) class vector from a binary (dummy) matrix.

** INPUT
Y = Dummy matrix. See 'koplsDummy()' for details.

** OUTPUT
classVect = The reconstructed integer class vector.
```

6.18 koplsRescale

```
function [scaleS]=koplsRescale(scaleS,varargin)


Scales a matrix based on pre-defined parameters from a scaling
object.

** INPUT
scaleS = An object containing scaling parameters
  (see 'koplsScale()').
varargin = If defined, this matrix will be scaled and returned.
Otherwise the original data set in the scaleS object will be
  scaled and returned.

** OUTPUT
scaleS = An object containing the following entries:
```

```
  centerType = 'mc' (mean-centering) or 'no' (no centering).
  scaleType = 'uv' (unit variance), 'pa' (pareto) or 'no'
      (no scaling).
  meanV = vector with mean values for all columns in X.
  stdV = vector with standard deviations for all columns in X.
  X = Scaled version of 'varargin', if defined, otherwise,
      scaled version of scaleS.X from input. Scaling is done
      according to 'centerType' and 'scaleType'.
```

6.19 koplsScale

```
function [scaleS]=koplsScale(X,centerType,scaleType)


Function for mean-centering and scaling of a matrix.

** INPUT
X = X matrix (to be mean-centered/scaled).
centerType = 'mc' for mean-centering, 'no' for no centering.
scaleType = 'uv' for unit variance scaling, 'pa' for Pareto
  scaling, 'no' for no scaling.

** OUTPUT:
scaleS = An object with the following properties:
  centerType = 'mc' or 'no'.
  scaleType = 'uv', 'pa' or 'no'.
  meanV = vector with mean values for all columns in X.
  stdV = vector with standard deviations for all columns in X.
  X = Original input matrix X, scaled according to 'centerType'
      and 'scaleType'.
```

6.20 koplsScaleApply

```
function [scaleSA]=koplsScaleApply(X,scaleS)
```

```
Applies scaling from external scaling objects (see
'koplsScale()') on a matrix X. Returns the scaled matrix,
including scaling settings.

** INPUT
X = X matrix (to be scaled).
scaleS = An object containing scaling parameters
  (see 'koplsScale()').

** OUTPUT
scaleSA = An object with the following properties:
  centerType = 'mc' or 'no'.
  scaleType = 'uv', 'pa' or 'no'.
  meanV = vector with mean values for all columns in X.
  stdV = vector with standard deviations for all columns in X.
  X = Scaled version of 'X', scaled according to 'centerType'
      and 'scaleType'.
```

6.21 koplsSensSpec

```
function [sensvec, specvec, classvec, tot_sens,meanSens,
meanSpec]=koplsSensSpec(v, m)
```

```
Calculates sensitivity and specificity in a class-wise fashion.

** INPUT
v = row vector of true class assignments (template).
m = matrix (or row vector) of class assignments to be compared.

** OUTPUT
sensvec = sensitivity for each class.
specvec = specificity for each class.
classvec = the class identifier corresponding to each column in
  sensvec and specvec.
tot_sens = total sensitivity.
meanSens = mean sensitivity over all classes.
meanSpec = mean specificity over all classes.
```

6.22 koplsDemo

```
This script contains a demonstration of the functionality in the
'kopls' package using a simulated data set. The data set is
represented by 1000 spectral variables from two different classes
and is available in the 'koplsDemo.mat' workspace. The
demonstration essentially consists of two main steps.

The first step is to demonstrate how K-OPLS handles the
model evaluation (using cross-validation), model building and
subsequent classification of external data from a non-linear data
set. The second step is to demonstrate how K-OPLS works in the
presence of response-independent (Y-orthogonal) variation, using
the same data set but with a strong systematic class-specific
disturbance added.

** THE 'koplsDemo.mat' WORKSPACE
The koplsDemo.mat workspace contains the following objects:
  Xtr = The training data matrix, with 400 observations and
      1000 spectral variables.
  Xte = The test data matrix, with 400 observations and
      1000 spectral variables.
  Xtro = Same data as 'Xtr', but with class-specific systematic
      noise added.
  Xteo = Same data as 'Xte', but with class-specific systematic
      noise added.
  Ytr = A binary matrix of class assignments for the training data.
  Yte = A binary matrix of class assignments for the test data.
  class1 = A vector of indicies of the samples in class #1.
  class2 = A vector of indicies of the samples in class #2.


** INSTRUCTIONS
1) Make sure that all 'kopls*.m' files are in the current path.
2) Run 'koplsDemo'.
```

References

[1] Rantalainen M, Bylesjö M, Cloarec O, Nicholson JK, Holmes E and Trygg J. **Kernel-based orthogonal projections to latent structures (K-OPLS)**. *J Chemometrics* 2007; 21:376-385.
